# Chemical Profiling and Assessment of Analgesic and Anti-Inflammatory Activity of *Ammoides verticillata* Essential Oil: In Vitro, In Vivo, and In Silico Studies

**DOI:** 10.3390/ph18050635

**Published:** 2025-04-27

**Authors:** Imene Derardja, Redouane Rebai, Fethi Benbelaïd, Luc Jasmin, Abdennacer Boudah, Mohammed Esseddik Toumi, Salsabil Mebarki, Fethi Farouk Kebaili, Leila Bellebcir, Alain Muselli

**Affiliations:** 1 Department of Biology, Faculty of Exact Sciences and Natural Life Sciences, Mohamed Khider University, P.O. Box 145 RP, Biskra 07000, Algeria; imene.derardja@univ-biskra.dz (I.D.); salsabil.mebarki@univ-biskra.dz (S.M.); leila.bellebcir@univ-biskra.dz (L.B.); 2 Laboratory Promotion of Innovation in Agriculture in Arid Regions (PIARA), Department of Biology, Faculty of Exact Sciences and Natural Life Sciences, Mohamed Khider University, P.O. Box 145 RP, Biskra 07000, Algeria; 3 Laboratory of Biotechnology, National Higher School of Biotechnology, Constantine 25000, Algeria; a.boudah@ensbiotech.edu.dz; 4 Laboratory of Applied Microbiology to Agrifood, Biomedical and Environment (LAMAABE), Aboubekr Belkaïd University, Imama Biomedical Complex P.O. Box 119, Tlemcen 13000, Algeria; fethi.benbelaid@univ-temouchent.edu.dz; 5 Department of Biology, Faculty of Science and Technology, Ain Temouchent University, Road of Sidi Bel Abbes BP 284, Ain Temouchent 46000, Algeria; 6 Department of Oral and Maxillofacial Surgery, University of California, San Francisco, 707 Parnassus Ave Suite D-1201, San Francisco, CA 94143, USA; luc.jasmin@ucsf.edu; 7 Laboratory of Microbiological Engineering and Application, Biochemistry and Molecular and Cellular Biology Department, Faculty of Nature and Life Sciences, University of Mentouri Brothers Constantine 1, Constantine 25017, Algeria; mohammed.es.seddik.toumi@umc.edu.dz (M.E.T.); kff.fethi@gmail.com (F.F.K.); 8 University of Corsica, UMR CNRS SPE 6134, Laboratory of Natural Products Chemistry, Campus Grimaldi, BP 52, 20250 Corte, France; muselli_a@univ-corse.fr

**Keywords:** *Ammoides verticillata*, essential oil, anti-inflammatory effects, analgesic activity, COX-2 inhibition, molecular docking

## Abstract

**Background/Objectives**: Essential oils are increasingly recognized for their therapeutic potential, yet *Ammoides verticillata* essential oil (AVEO) remains relatively unexplored, particularly for its anti-inflammatory and analgesic properties. This study aimed to profile AVEO’s chemical composition and evaluate its antioxidant, anti-inflammatory, and analgesic effects, with a focus on its novel pharmacological actions. **Methods**: The chemical composition of AVEO was determined using GC-MS analysis, and antioxidant capacity was assessed through in vitro assays. Furthermore, the anti-inflammatory potential was investigated using a carrageenan-induced paw edema model in rats, complemented by the inhibition assays of cyclooxygenase (COX) enzymes. The analgesic effects were evaluated through acetic acid-induced writhing and tail immersion tests. Additionally, a computational study was performed to explore the binding affinity of AVEO’s major constituents to COX-2. **Results**: GC-MS analysis revealed a rich monoterpene profile dominated by carvacrol (32.51%). It was found that AVEO exhibited significant antioxidant activity. Similarly, in vivo, AVEO showed significant anti-inflammatory effects, achieving a percentage inhibition of 52.23% at 200 mg/kg, comparable to diclofenac, along with potent COX-2 inhibition observed (IC_50_ = 1.51 ± 0.20, SI = 5.56). Moreover, analgesic tests demonstrated dose-dependent pain relief, in which the dose of 200 mg/kg significantly prolonged tail latency to 14.00 ± 1.45 s and markedly reduced abdominal constriction to 21.17 ± 1.62. Computational analysis further corroborated the high binding affinity of carvacrol and thymol with COX-2 (−7.381 and −6.939 Kcal/mol, respectively). **Conclusions**: These findings underscore AVEO’s potential as a promising therapeutic agent for managing inflammation and pain.

## 1. Introduction

Inflammation is a vital defense mechanism of the immune system, naturally activated in response to disruption in the body’s homeostasis, whether due to infectious agents, chemical irritants, physical injury, or biological factors [1,2]. While essential for host defense and tissue repair, inflammation can act as a biological double-edged sword. Acute inflammation is an orchestrated process that eliminates harmful stimuli and restore homeostasis. However, when inflammation became chronic or dysregulated, it can serve as a pathological trigger [3,4,5], contributing to the progression of complex diseases such as arthritis [6], cardiovascular disorders [7], neurodegenerative conditions [8], and even cancer [2].

Pain, an unpleasant neurological response signaling potential tissue damage, is a frequent complication of chronic inflammation. It acts as an early warning system, warning the body of harm [9,10]. However, in chronic conditions, pain persists due to the intense release of pro-inflammatory mediators such as tumor necrosis factor-alpha (TNF-α), interleukin-1 beta (IL-1β), interleukin-6 (IL-6), nitric oxide (NO), and prostaglandin E2 (PGE2). These mediators sensitize nociceptors, amplifying pain perception and creating a feedback chain between inflammation and pain, contributing to disease progression [9,10]. PGE2 acts as a crucial inflammatory mediator, synthesized from specific cell membrane fatty acids through reactions catalyzed by the cyclooxygenase enzyme. This enzyme is present in two isoforms, COX-1 and COX-2. Under typical circumstances, COX-1 is consistently present in cells, leading to the synthesis of protective compounds for the stomach. Conversely, in pathological circumstances, pro-inflammatory substances such as cytokines trigger the production of COX-2. Therefore, when there is inflammation or tissue injury, COX-2 levels rise [11,12].

Non-steroidal anti-inflammatory drugs (NSAIDs) have long been the standard for managing pain, fever, and inflammation by inhibiting COX enzymes [11]. However, the traditional non-selective NSAIDs frequently cause gastrointestinal complications [13]. Although newer COX-2-selective NSAIDs offer some improvement, they are associated with cardiovascular side effects, including myocardial infarction and stroke, due to imbalances in vascular prostacyclin (PGI2) production, which may disrupt normal homeostasis [12,14]. These limitations highlight the need for safer anti-inflammatory agents with selective COX-2 inhibition.

In this context, medicinal plants have gained attention as a rich source of bioactive compounds with fewer side effects compared to their synthetic counterparts [15]. Among these candidates, *Ammoides verticillata* stands out as a compelling subject for investigation. Commonly found in West Algeria, this aromatic medicinal herb has been extensively utilized in traditional medicine for respiratory issues, headaches, gastrointestinal disorders, and renal infections [16,17]. Moreover, *A. verticillata* has demonstrated diverse properties, such as antioxidant, anti-microbial [18,19], antidiabetic [20,21], antihypertensive, and relaxant activities [22,23]. Previous studies have extensively explored the essential oil derived from *A. verticillata*, establishing its anti-microbial, antioxidant, and anti-proliferative capacities [22,24]. It has also demonstrated in vitro inhibitory effects on key enzymes such as α-amylase, xanthine oxidase, acetylcholinesterase, and butyrylcholinesterase [25,26]. Furthermore, AVEO has shown promising in vitro anti-inflammatory activity, particularly through 5-lipoxygenase (5-LOX) inhibition [25].

Despite promising findings, a notable gap remains in the literature regarding the analgesic and anti-inflammatory properties of AVEO, particularly its COX-2 inhibition. This study aims to address this gap by providing the first comprehensive investigation of the in vivo analgesic and anti-inflammatory effects of AVEO, alongside in silico studies to explore its molecular interactions with COX-2. Furthermore, an acute toxicity assessment was conducted to establish safe and effective in vivo dosing, bridging the lack of previous toxicological studies on AVEO.

## 2. Results

### 2.1. Phytochemical Chemical Profiling of A. verticillata Essential Oil: Composition Analysis and Yield

The essential oil derived from *Ammoides verticillata* aerial parts yielded 2% (*w*/*w*) from 100 g of plant material following a three-hour steam-distillation process. The extracted oil was characterized by a light-yellow color and a strong, distinctive aroma. The identified constituents, along with their retention times, relative percentages, and corresponding chemical classes, are summarized in Table 1.

Gas chromatography–mass spectrometry (GC-MS) analysis identified 18 distinct compounds, collectively accounting for 96.58% of the total oil composition. The essential oil was predominantly characterized by monoterpene hydrocarbons, which represented 49.92% of its total composition. Oxygenated monoterpenes, on the other hand, also represented a significant portion of the oil’s composition, at about 46.66%. Among the monoterpene hydrocarbons, the most abundant compounds included gamma-terpinene (17.59%), para-cymene (15.45%), and limonene (15.22%). The oil’s chemotype was determined to be carvacrol, an oxygenated monoterpene that constituted 32.51% of the total composition. Additionally, thymol, another phenolic monoterpene, was identified as a significant constituent, constituting 12.49% of the overall composition. Trace amounts of other compounds were also detected, including myrcene (0.89%), alpha–pinene (0.77%), terpinen-4-ol (0.68%), and beta–pinene (0.51%).

### 2.2. Antioxidant Potential

Various assays were used to assess the antioxidant potential of AVEO, each based on a different reaction mechanism. The findings are summarized in Table 2. AVEO showed moderate DPPH^•^ radical scavenging activity, with an IC_50_ value of 83.11 ± 0.34 µg/mL. However, this activity was significantly lower (*p* < 0.001) compared to that of the natural antioxidants, ascorbic acid and α-tocopherol, which exhibited IC_50_ values of 3.45 ± 0.16 and 4.36 ± 0.68 µg/mL, respectively.

In the ABTS test, AVEO demonstrated very good scavenging activity against ABTS**^•+^** radicals, with an IC_50_ value of approximately 3.52 ± 0.02 µg/mL. However, this efficacy was considerably higher than that of ascorbic acid and α-tocopherol (*p* < 0.001), which showed IC_50_ values of 7.43 ± 0.29 and 9.59 ± 0.08 µg/mL, respectively. In addition, AVEO has shown a low total antioxidant capacity, with a concentration around 7.689 ± 0.082 µg AAE/mg EO, compared to the standard antioxidant α-tocopherol, which showed a higher value of 75.640 ± 0.075 µg AAE/mg EO.

From the FRAP assay, AVEO showed acceptable reducing power by reducing ferric iron (Fe^3+^) in solution with an IC_50_ value around 57.81 ± 1.88 µg/mL. Reference antioxidants, ascorbic acid and α-tocopherol, showed higher reducing activities (*p* < 0.001) with IC_50_ values of 24.62 ± 1.20 and 37.39 ± 2.14 µg/mL, respectively.

### 2.3. Acute Toxicity Assessement

In the evaluation of the acute toxicity of AVEO, mortality and visible toxicity signs were monitored over a 14-day observation period. No deaths occurred in the control group or in the group receiving 1000 mg/kg of EO. In the group treated with 2000 mg/kg, two out of six rats died within 48 h post-administration. Signs of toxicity, including hyper-salivation, hypo-activity, and abdominal pain, were observed shortly after administration in the 2000 mg/kg group, but these symptoms disappeared within 24 h. The remaining animals in this group and other animals remained stable without further mortality or significant adverse effects throughout the study. Furthermore, the results of the acute toxicity tests for GOT, GPT, ALP, lipid profile (triglycerides and total cholesterol), uric acid, and creatinine are listed in Table S1. The biochemical parameters in the treated groups, which received the acute dose of AVEO (1000 mg/kg or 2000 mg/kg), showed no significant changes compared with the control group.

### 2.4. In Vivo Anti-Inflammatory Effects

**Figure 1:**
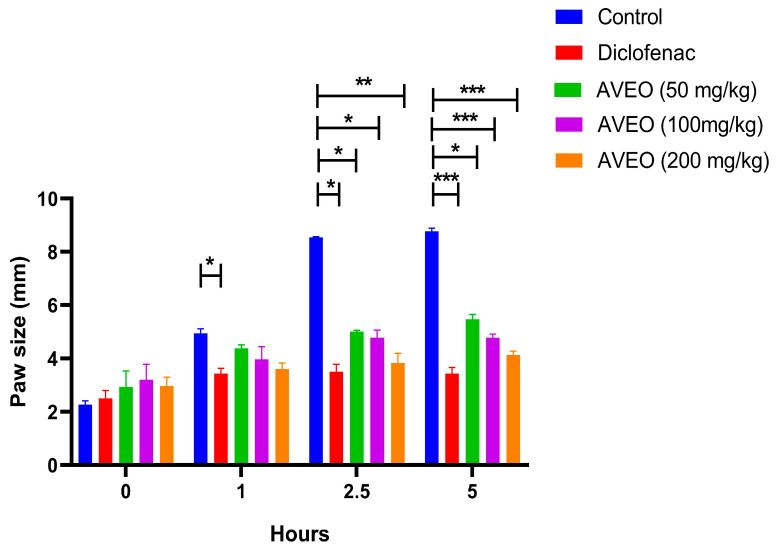
Paw edema progression over time following oral administration of AVEO and diclofenac (20 mg/kg) in carrageenan-induced model. Each value represents mean ± SEM analyzed by ANOVA test followed by post hoc Tukey’s test (* *p* < 0.05, ** *p* < 0.01, and *** *p* < 0.001).

**Figure 2:**
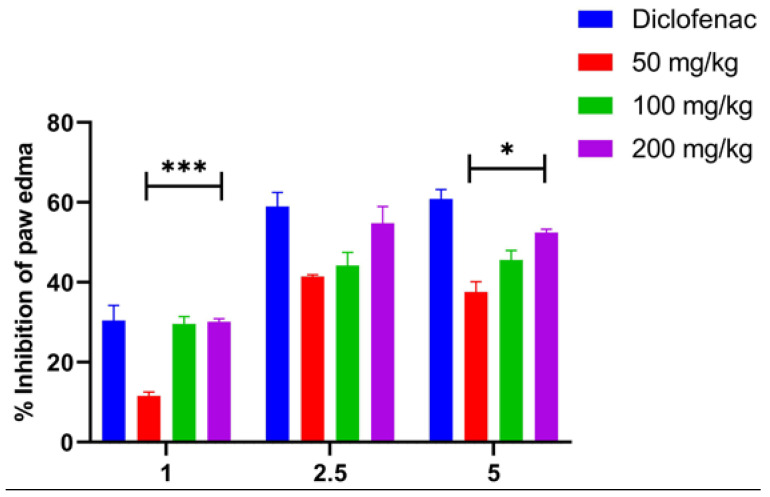
Efficacy of oral administration of AVEO and diclofenac (20 mg/kg) in reducing carrageenan-induced paw edema in rats. Each value represents mean ± SEM analyzed by ANOVA test followed by post hoc Tukey’s test (* *p* < 0.05 and *** *p* < 0.001).

The intraplantar injection of carrageenan induced a significant, time-dependent increase in paw size, with the most pronounced swelling observed at 2.5 h post exposure (Figure 1). Compared to the control group, pretreatment with AVEO (50, 100, and 200 mg/kg) significantly reduced carrageenan-induced paw edema from 2.5 h onwards (*p* < 0.05, *p* < 0.05, and *p* < 0.01, respectively), achieving maximum inhibition rates of 41.40%, 44.15%, and 54.76%, respectively. Similarly, its development was significantly reduced after diclofenac treatment (58.95%) (Figure 1 and Figure 2).

By the fifth hour, both diclofenac and AVEO, at 200 mg/kg, demonstrated potent efficacy in reducing paw edema compared to the control group. Their inhibitory effects on edema progression were statistically comparable (*p* > 0.05), with inhibition rates of 60.86% and 52.23%, respectively (Figure 1 and Figure 2).

A higher AVEO dose (200 mg/kg) tends to cause a greater paw volume reduction (52.23%) than 50 mg/kg (41.44%), indicating that the effect is dose dependent (Figure 2).

### 2.5. Analgesic Properties

#### 2.5.1. Tail Immersion Test: Central Anti-Nociceptive Activity

**Figure 3:**
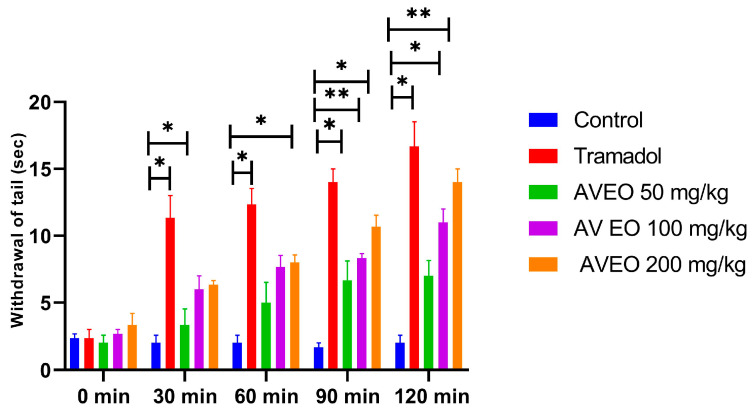
Tail withdrawal latency of rats following treatment with *A. verticillata* essential oil compared to tramadol. Results are expressed as mean ± SEM and analyzed using ANOVA test followed by post hoc Tukey’s test (* *p* < 0.05 and ** *p* < 0.01).

The central analgesic activity of AVEO was assessed using the tail immersion test, with tramadol serving as the reference drug. The results indicate that pretreatment with AVEO and tramadol produced significant analgesic responses, which became evident 30 min post administration, with peak efficacy at 120 min (Figure 3).

The AVEO presented an anti-nociceptive effect in a dose-dependent manner at 100 and 200 mg/kg (Figure 3).

For the dose of 100 mg/kg of AVEO, a significant increase in withdrawal time (6.66 s at 90 min, and 7.00 s at 120 min, respectively) was observed when compared to the control group (Figure 3).

Specifically, the administration of AVEO at 200 mg/kg significantly prolonged tail withdrawal latency during 30 min, 60 min, and 90 min, reaching 14.00 ± 1.45 s at 120 min (Figure 3). Likewise, tramadol demonstrated a sustained and significant (*p* < 0.05) pain-relieving effect across all observation intervals, with maximum efficacy recorded at 120 min post treatment. Interestingly, the analgesic action of AVEO at 200 mg/kg was comparable to that of tramadol at the 120 min time point (Figure 3), suggesting a potent central analgesic effect.

#### 2.5.2. Acetic Acid-Induced Writhing Test: Peripheral Anti-Nociceptive Activity

**Figure 4:**
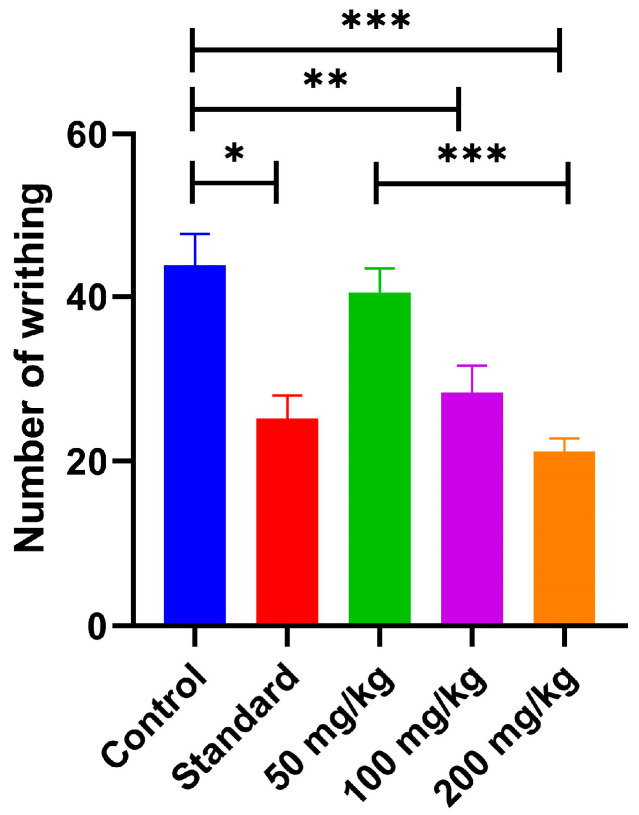
Analgesic effect of oral pretreatment with AVEO and diclofenac (15 mg/kg) on acetic acid-induced writhing in rats. Data are presented as mean ± SEM and analyzed using two-way ANOVA test followed by post hoc Tukey’s test (* *p* < 0.05, ** *p* < 0.01, and *** *p* < 0.001).

The peripheral analgesic activity of AVEO was evaluated using the acetic acid-induced abdominal writhing test. AVEO administration led to a dose-dependent and statistically significant reduction in the number of abdominal constrictions (Figure 4). The injection of acetic acid significantly increased the number of writhing in the control group (43.8 ± 3.80) (Figure 4). Pretreatment with AVEO at the dose of 50 mg/kg showed no significant effect. However, the administration of 100 mg/kg and 200 mg/kg doses reduced the writhing number to 28.33 ± 3.30 and 21.17 ± 1.62, respectively, compared to the control group (Figure 4). In comparison, the reference drug, diclofenac sodium (15 mg/kg), also produced a significant decrease in abdominal constrictions. The observed inhibitory effects suggest that AVEO exhibits potent peripheral analgesic activity, with efficacy comparable to that of diclofenac sodium.

### 2.6. In Vitro COX-1 and COX-2 Inhibition Assays

The in vitro anti-inflammatory activity of AVEO was evaluated by assessing its inhibitory effects on cyclooxygenase enzymes (COX-1 and COX-2), with diclofenac sodium and celecoxib serving as reference inhibitors. After determining the half-maximal inhibitory concentrations (IC_50_), the selectivity index (SI) for COX-2 inhibition was calculated using the following formula:

SI = IC_50_ (COX-1)/IC_50_ (COX-2)

As presented in Table 3, AVEO exhibited moderate COX-1 inhibitory activity, evidenced by an IC_50_ value of 8.39 ± 0.50 µg/mL. In comparison, both reference drugs showed stronger COX-1 inhibition, as reflected by lower IC_50_ values of 4.02 ± 0.60 µg/mL for diclofenac (*p* < 0.001) and 3.30 ± 0.47 µg/mL for celecoxib (*p* < 0.001).

Regarding COX-2 inhibition, AVEO demonstrated notable inhibitory activity, achieving an IC_50_ of 1.51 ± 0.20 µg/mL, which was comparable to diclofenac (1.06 ± 0.38 µg/mL, *p* > 0.05). As expected, celecoxib exhibited the highest potency, with an IC_50_ of 0.17 ± 0.04 µg/mL, confirming its strong selectivity.

The selectivity index (SI), which reflects the preferential inhibition of COX-2 over COX-1, was in the order of 5.56 for AVEO, indicating favorable COX-2 selectivity. In comparison, diclofenac displayed lower SI (3.79), whereas celecoxib exhibited the highest selectivity (19.41). While celecoxib remained the most selective COX-2 inhibitor, AVEO’s SI still suggests preferential COX-2 inhibition, reinforcing its anti-inflammatory potential.

### 2.7. Molecular Insights into COX-2 Inhibition: Computational Findings

#### 2.7.1. Molecular Docking and MMGBSA Findings

The computational analysis was conducted to evaluate the anti-inflammatory potential of AVEO-derived compounds, focusing on their interaction with COX-2. The root mean square deviation (RMSD) between the crystallographic pose of the native inhibitor and the re-docked conformation was determined to be less than 2 Å (RMSD = 1.431 Å) (Figure S1), confirming the accuracy of the docking approach [27].

The docking results revealed that AVEO compounds exhibited varied binding affinities toward the COX-2 enzyme, with glide scores ranging from −7.381 to −4.239 Kcal/mol (Table S2).

Among the main compounds, carvacrol and thymol demonstrated the strongest affinities, with glide scores of −7.381 Kcal/mol and −6.939 Kcal/mol, respectively (Table 4). However, carvacrol showed a more favorable binding energy (−51.50 Kcal/mol) compared to thymol (−42.35 Kcal/mol), suggesting better stabilization within the COX-2 active site.

Gamma-terpinene and para-cymene also displayed considerable affinities, with glide scores of −6.370 Kcal/mol and −6.255 Kcal/mol, respectively, and corresponding binding energies of −61.64 Kcal/mol and −46.95 Kcal/mol, respectively (Table 4). Interestingly, despite limonene showing a moderate score of −5.666 Kcal/mol, it presented a relatively high binding energy of −53.05 Kcal/mol (Table 4).

In comparison, diclofenac, the reference drug, exhibited the highest glide score (−7.837 Kcal/mol), reflecting its strong binding affinity, yet its binding energy (−34.62 Kcal/mol) was lower than those of AVEO-derived compounds.

Post-docking visualization confirmed that all tested compounds were well-accommodated in the COX-2 active site, interacting with key residues such as Tyr385, Val349, Trp387, and Ser530, similar to diclofenac (Table 5). Detailed molecular interactions are presented for the major constituents, with comprehensive data for the minor constituents available in the Supplementary Material (Table S3).

Carvacrol and thymol, the top-performing ligands, showed distinct interactions that enhanced their binding affinities (Figures S2 and S3). Both compounds formed single hydrogen bond with Ser350, measuring 1.80 Å and 1.93 Å, respectively, alongside several hydrophobic interactions with Leu352, Phe518, Val349, Ala527, Val523, Met522, Phe381, Leu384, Tyr385, Trp387, and Tyr348 (with thymol not interacting with Tyr348). Pi–Pi interactions were observed with Phe518 (carvacrol) and Trp387 (thymol) (Table 5). In contrast, diclofenac’s binding was primarily stabilized by hydrophobic interactions with residues such as Tyr355, Leu352, Val349, Tyr348, Phe518, Tyr385, and Trp387, along with Pi–Pi stacking with Tyr355 (Table 5). The similarities in interaction patterns between AVEO-derived compounds and diclofenac support the potential of AVEO.

#### 2.7.2. ADME Prediction

Evaluation of selected ADME properties of the main AVEO constituents revealed that selected compounds adhere to both Lipinski’s and Veber’s rules, indicating favorable oral bioavailability (Table S4). While compliance with these rules is a positive indicator, it should be noted that this alone does not guarantee the pharmaceutical suitability of the compounds.

The lipophilicity, measured as QPlog Po/w, was within the recommended limits for all compounds (Log *p* < 5 according to Lipinski’s rule). Regarding topological polar surface area values (TPSA), carvacrol and thymol showed values within the acceptable range (TPSA < 140 Å, as per Veber’s rule). However, gamma-terpinene, para-cymene, and limonene showed TPSA values of zero (Table S4).

In terms of absorption, the compounds displayed high intestinal absorption (Table S2), indicating that they are likely to be absorbed upon oral administration, which suggests their potential for effective oral bioavailability. Furthermore, all the compounds showed positive activity on the central nervous system (CNS) and the ability to cross the blood–brain barrier, as indicated by their predictive QPlog BB values (Table S5). In addition, they were identified as non-blockers of hERG potassium channels, suggesting a minimal risk for adverse cardiac effects, a key consideration in drug development, particularly in the search for new COX-2 inhibitors.

#### 2.7.3. Molecular Dynamics Simulations

To conduct a more in-depth analysis, the best docked complexes were further subjected to MD simulations. This step aimed to assess the stability and the flexibility of the hit compounds in the COX-2 binding pocket as a function of time. To this end, an MD simulation of carvacrol and thymol was carried out for 100 ns in a more realistic environment that accounts for protein and ligand flexibility, as well as the solvent effects. The root means square deviation (RMSD) and root square fluctuation (RMSF) were used to analyze and evaluate the protein–ligand complex stability. In which, the RMSD provides a measure of stability, while RMSF assesses the flexibility of the protein’s amino acid residues, particularly those located in the active site.

**Figure 5:**
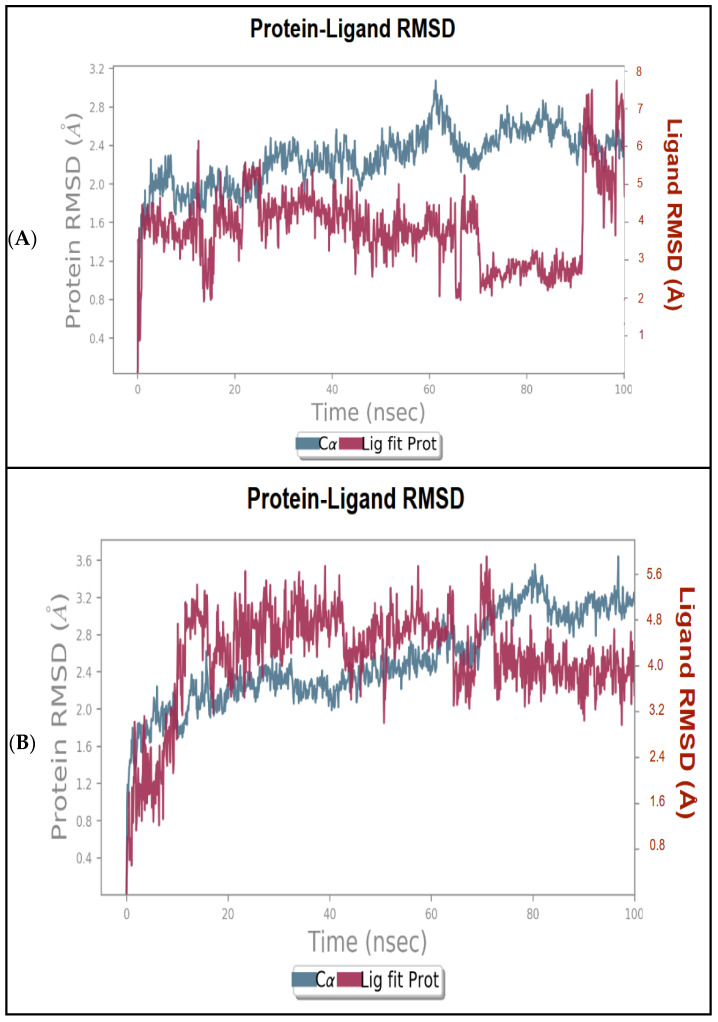
RMSD analysis of lead compounds in complex with COX-2. (**A**) RMSD plot of carvacrol-COX-2 complex. (**B**) RMSD plot of thymol–COX-2 complex.

Analysis of RMSD plots (Figure 5A) revealed that the carvacrol–COX-2 complex demonstrated acceptable stability throughout the first 50 ns of the simulation. During this period, the protein backbone showed a low fluctuation (1.6–2.4 Å), indicating structural stability. The ligand also remained stable, well-positioned within the binding pocket, with minimal deviations, suggesting notable molecular interactions. Beyond 50 ns, the ligand began to display moderate fluctuations, reflecting structural rearrangement. However, after 80 ns, the ligand RMSD increased significantly, reaching approximately 7.5 Å, indicative of diffusion behavior or partial dissociation from the active site. Despite this, the protein structure remained relatively stable throughout the simulation, with no substantial conformational deviations. After 90 ns, the complex re-stabilized for the remainder of the simulation.

For the thymol–COX-2 complex, the system demonstrated greater stability, remaining well maintained for the first 75 ns (Figure 5B). Throughout this timeframe, thymol exhibited good binding; nevertheless, it showed slight fluctuations with an RMSD ranging from 0.4 to 2.8 Å for the protein, and from 0.8 to 5.6 Å for the ligand. After 75 ns, thymol displayed a diffusion behavior for the rest of the simulation, yet the average RMSD fluctuations for both protein and ligand remained relatively low until the end of the simulation.

In this context it is important to note that variations in the order of 1 to 3 Å are perfectly acceptable for small globular proteins. The slight RMSD fluctuations of the carvacrol–COX-2 and thymol–COX-2 complexes at the end of the simulation time indicate that the complexes have reached more or less a stable state, i.e., the system tends to reach equilibrium.

**Figure 6:**
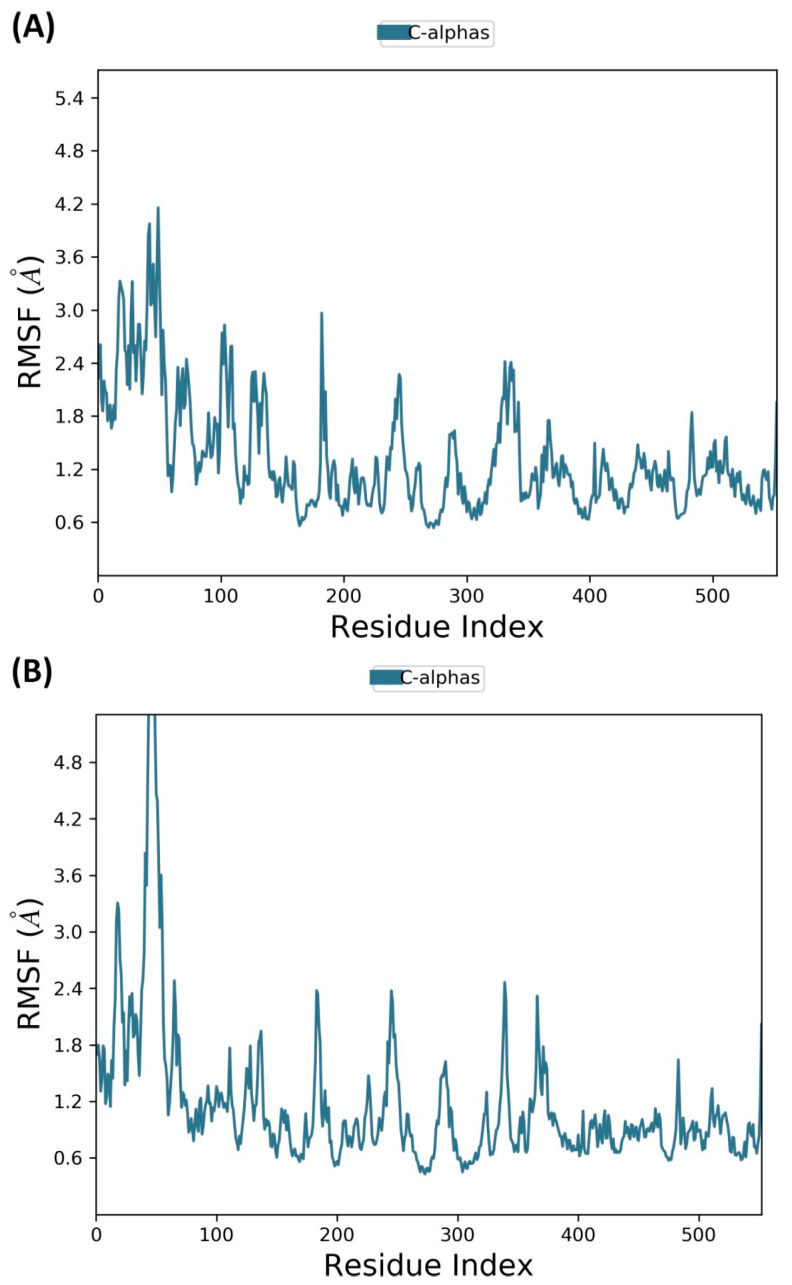
RMSF analysis of COX-2 protein with the selected compounds. (**A**) RMSF graph of carvacrol–COX-2 complex. (**B**) RMSF graph of thymol–COX-2 complex.

In parallel, RMSF analysis was performed to identify protein regions with the highest flexibility during the simulation. As illustrated in Figure 6, the most important fluctuations were observed in the N-terminal region, with a maximum RMSF value equal to 4.2 Å for carvacrol-COX-2 complex (Figure 6A) and greater than 4.8 Å for thymol-COX-2 complex (Figure 6B). These fluctuations primarily occurred in residues distant from the ligand-binding pocket (first 200 residues). In contrast, the key residues within the active site, which directly interact with the ligands (positions between 350 and 530), remained relatively stable, exhibiting only minor fluctuations throughout the simulation.

**Figure 7:**
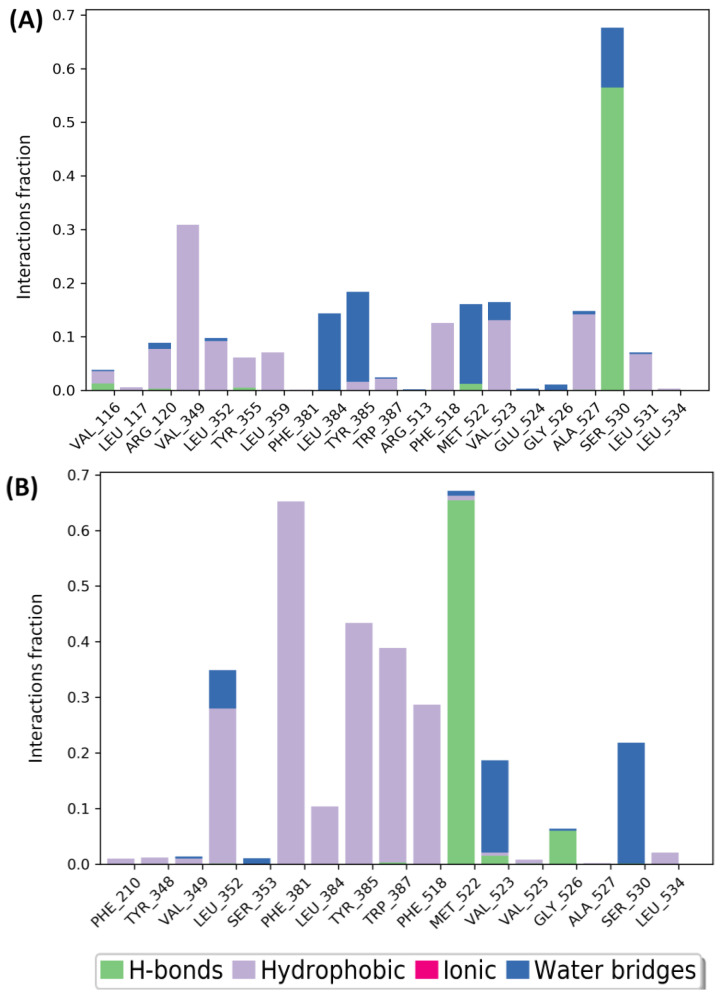
Histograms of protein–ligand interactions throughout the simulation period. (**A**) Carvacrol–COX-2 complex contacts. (**B**) Thymol–COX-2 complex contacts.

The molecular interactions established between COX-2 and the top hits for 100 ns were analyzed using protein–ligand contact histograms (Figure 7). Both compounds demonstrated consistent interactions throughout the simulation, with carvacrol maintaining a hydrogen bond with Ser530 for over 50% of the simulation time (Figure 7A), while thymol formed a hydrogen bond with Met522 (Figure 7B), likely due to conformational fluctuations of both the ligand and protein. Additionally, both compounds established hydrophobic interactions with almost conserved residues, as observed in the docking analysis. Overall, these results suggest that both candidates showed a stable tendency to bind with key residues in the COX-2 active site.

## 3. Discussion

Given the growing demand for natural, safer alternatives to synthetic drugs for the management of inflammation and pain, AVEO has emerged as a promising candidate for the development of plant-based anti-inflammatory and analgesic therapies. Through a combination of in vivo and in vitro models, we have successfully elucidated AVEO’s anti-inflammatory and anti-nociception actions. Furthermore, our findings highlight how its key constituents, carvacrol and thymol, modulate critical inflammatory pathways, notably through COX-2 inhibition, as demonstrated by in silico analysis. Utilizing this approach, we provide insights into AVEO’s pharmacological potential and its possible mechanisms of action, particularly its COX-2 inhibitory effects.

The GC-MS analysis of AVEO revealed a chemical profile largely consistent with existing literature on this species. Monoterpenes, both hydrocarbons and oxygenated forms, dominated the oil’s composition, accounting for 96.58% of the total content. The chemotype was identified as carvacrol, which constituted 32.51%, with *gamma*-terpinene, *para*-cymene, limonene, and thymol as major constituents, collectively representing 93.26% of the oil’s overall composition.

Comparison with previous studies indicates a high degree of consistency in the primary constituents of AVEO, particularly in the dominance of carvacrol. Studies on AVEO sourced from Algeria have similarly identified carvacrol as the main compound, aligning with our findings [16,23,28]. However, some reports have noted chemotypic variability, with thymol being the dominant compound in certain samples [29,30], possibly due to environmental factors such as geography, climate, and soil composition.

Furthermore, studies from Morocco reported carvacrol as the principal component of AVEO, further corroborating our findings. In contrast, Tunisian studies have shown more varied results, with some identifying thymol as the major compound [31,32], whereas a study by [25] reported peril aldehyde as the most abundant compound for the first time. Despite these regional variations in the predominant compound, the presence of key constituents such as *gamma*-terpinene, *para*-cymene, limonene, carvacrol, and thymol appears to be a common feature of AVEO, regardless of its geographical origin.

The assessment of AVEO’s antioxidant potential revealed good efficacy, with varied effectiveness depending of the specific assay and chemical reaction mechanisms. To gain a comprehensive understanding of its antioxidant effects, we employed both single electron transfer (SET)-based and mixed-mode assays (HAT/SET), which incorporate both hydrogen atom transfer (HAT) and SET mechanisms. AVEO displayed contrasting tendencies in its ability to scavenge DPPH**^•^** and ABTS**^•+^** radicals. While its ability to quench DPPH**^•^** was moderate, it demonstrated remarkable scavenging capacity against ABTS**^•+^** radicals, suggesting varied kinetic behavior with these model radicals. The observed 23-fold difference in scavenging capacity can be attributed to the distinct nature, structure, and reactivity of the radicals. While both DPPH**^•^** and ABTS**^•+^** are stable radicals, ABTS**^•+^** is known to be more reactive, and electron transfer from antioxidants and ABTS**^•+^** occurs much faster than in the DPPH assay. This difference is likely due to steric hindrance of the DPPH radical site, which makes interaction with antioxidants more challenging [33,34].

These findings align with previous studies, including the research by [29], which demonstrated the antioxidant potential of AVEO extracted from leaves and flowers collected from various regions in Algeria. Their study reported scavenging activity against DPPH**^•^** radicals, with IC_50_ values ranging from 106 to 380 µg/mL for the flowers and from 73 to 338 µg/mL for the leaves. Furthermore, the EOs displayed inhibitory activity against ABTS**^•+^** radicals, with IC_50_ values ranging from 2 to 7 µg/mL for the flowers and from 2 to 20 µg/mL for the leaves. Similarly [26], reported that AVEO scavenged DPPH**^•^** and ABTS**^•+^** radicals, with IC_50_ values of 139.28 ± 0.48 and 1.82 ± 0.05 µg/mL, respectively. These results are consistent with our observations, further supporting the conclusion that AVEO contains compounds capable of mitigating oxidative stress through radicals scavenging.

In the FRAP assay, AVEO displayed a dose-dependent ability to reduce ferric iron (Fe^3+^) to ferrous iron (Fe^2+^), indicating its capacity to donate electrons, another important mechanism of antioxidant defense. However, in the total antioxidant capacity assay, the oil’s performance was moderate compared to α-tocopherol, suggesting that higher concentrations may be required to match the potency of well-established antioxidants like vitamin E.

Overall, the results demonstrated that AVEO possesses considerable antioxidant activity, primarily attributed to its key components, carvacrol and thymol, both of which are well known for their antioxidant properties [35,36]. These properties are likely due to the presence of hydroxyl groups, which can act as hydrogen donors, and the delocalization of electrons within their aromatic rings [37,38].

Before proceeding with in vivo studies, toxicological evaluation is a fundamental step in pharmacological research to ensure the safety of tested compounds or mixtures. For this reason, the acute oral toxicity of AVEO was assessed to establish appropriate dosing strategies. This evaluation is crucial not only for understanding AVEO’s potential impact on biological systems but also for ensuring the well-being of experimental subjects. Based on the observed mortality response at the tested doses, AVEO is classified as category 5 (2000–5000 mg/kg) under the globally harmonized system of classification and labeling of chemicals (GHS), which correspond to substances with relatively low acute toxicity [39]. This classification suggests that AVEO may be considered safe at lower doses. Moreover, the absence of significant alterations in biochemical markers related to hepatic and renal functions, as well as lipid metabolism, further supports the safety of AVEO. These results suggest that, under the conditions of this study, AVEO does not affect liver or kidney functions. Consequently, doses of 50 mg/kg, 100 mg/kg, and 200 mg/kg were selected for in vivo studies, ensuring a balance between safety and efficacy, as even small increases in dosage can significantly impact survival.

Following these assessments, the anti-inflammatory effects of AVEO were evaluated in vivo, focusing on its potential to attenuate acute inflammation. The carrageenan-induced paw edema model, a well-established method for screening orally active anti-inflammatory agents, was employed to investigate AVEO’s effects. This model is a biphasic process in nature, mediated by distinct molecular and cellular events, where the injection of carrageenan, a sulfated polysaccharide, triggers acute and local inflammatory response accompanied by the development of edema. The early phase (0–2.5 h) is driven by the release of vasoactive mediators such as histamine, serotonin, and bradykinin [40,41], leading to increased vascular permeability. The later phase (3–6 h) is characterized by sustained inflammation, primarily involving the overproduction of prostaglandins and pro-inflammatory cytokines, including NO, IL-1β, IL-6, and TNF-α [40,41,42].

Our findings revealed that AVEO significantly reduced paw edema in both phases of inflammation in a dose-dependent manner, with effects nearly comparable to those of diclofenac. This biphasic inhibition suggests that AVEO may interfere with vascular events during the early phase while simultaneously attenuating the cytokine-mediated processes of the late phase. Although further studies are necessary to elucidate the subjacent mechanisms, the inhibition of fluid extravasation in the early phase implies that AVEO may exert antagonistic effects on histamine, bradykinin, or serotonin, potentially modulating vascular permeability. Additionally, the reduction in edema in the late phase suggests its ability to down-regulate or inhibit pro-inflammatory mediators such as PGE2, NO, and TNF-α, highlighting its potential as an anti-inflammatory agent.

The significant anti-inflammatory effects of AVEO are likely attributed to its rich and diverse phytochemical composition, dominated by bioactive compounds such as carvacrol, thymol, limonene, para-cymene, and gamma-terpinene, all of which have been reported to exhibit various biological activities, particularly anti-inflammatory effects.

For instance, carvacrol, has been widely studied for its anti-inflammatory actions; [43] demonstrated that carvacrol significantly reduced paw edema and levels of IL-1β and PGE2 in mice models of acute inflammation, effects linked to the downregulation of COX-2 and IL-1β mRNA expression, along with an increase in the anti-inflammatory cytokine IL-10, which plays a crucial role in controlling excessive inflammatory responses. Several in vitro studies further confirmed that carvacrol inhibits the production of key pro-inflammatory cytokines such as TNF-α, IL-1β, and IL-6 [44,45], as well as nitric oxide [44]. It was also found to downregulate iNOS and COX-2 expression [46], and to suppress central inflammatory signaling pathways, notably ERK1/2 [43], NF-κB [44,45,46], and the phosphorylation of JNK and IKK [45]. In models of cardiac inflammation, carvacrol modulated key axes. Specifically, it inhibited the TLR4/MyD88/NF-κB pathway [47,48] and suppressed NLRP3 inflammasome activation [47,48,49], resulting in reduced levels of IL-18 [48,49], IL-1β [47,48,49], TNF-α [48], IL-6 [48], and GSDMD [48,49].

Furthermore, carvacrol has been identified as an activator of PPARα and PPARγ, nuclear transcription factors involved in the regulation of inflammation [50]. Evidence suggests that PPAR activation can suppress the inflammatory response by inhibiting of the NF-κB pathway [51]. Carvacrol also significantly suppresses LPS-induced COX-2 mRNA expression in human U937 macrophages in a PPARγ-dependent manner [50]. Additionally, recent findings demonstrated that carvacrol attenuated isoproterenol-induced myocardial ischemia via activation of PPARγ and Nrf2, along with downregulation of NLRP3 [52].

Similarly, thymol exhibits notable anti-inflammatory effects; [53] reported that thymol inhibited neutrophil elastase release, a key enzyme implicated in tissue damage during inflammation. Thymol also downregulated iNOS and COX-2 expression and suppressed pro-inflammatory cytokines such as TNF-α and IL-6 by blocking the phosphorylation of IκBα, NF-κB p65, and p38 [54]. Moreover, thymol demonstrated dose-dependent anti-inflammatory effects in an Alzheimer’s disease model, significantly reducing serum and hippocampal levels of IL-1, IL-6, TNF-α, and COX-2, with optimal effects at 80 mg/kg [55]. Additional studies highlighted that thymol exerts hepatoprotective effects by activating the Nrf2/HO-1 pathway and inhibiting NF-κB signaling [56]. In a stroke model, it improved neurological outcomes by reducing microglial activation and the expression of IL-6, IL-1β, and TNF-α, likely through inhibition of NF-κB [57]. Furthermore, thymol protected mice against LPS-induced liver injury by suppressing the NLRP3 inflammasome and reducing mRNA levels of TNF-α, IL-6, and IL-22 [58]. In vivo, it alleviated LPS-induced endometritis in mice and reduced TNF-α and IL-1β levels [59]. Complementary in vitro experiments in RAW264.7 macrophages confirmed that thymol dose-dependently suppressed the expression of TNF-α, IL-1β, iNOS, and COX-2, and it also inhibited the TLR4/NF-κB pathway [59].

Limonene, another prominent compound in AVEO, also contributes significantly to its anti-inflammatory activity. Previous studies [60] demonstrated that limonene reduced colonic mucosal damage in models of colitis by modulating iNOS and COX-2 expression. In LPS-induced jejunal injury, limonene exerted its effects by reducing IL-1β, TNF-α, COX-2, and iNOS levels while suppressing TLR4/NF-κB/AP-1 signaling and activating Nrf2 pathway [61]. In alcoholic liver disease models, limonene alleviated inflammation and oxidative stress through regulation of MAPK/Nrf2 and NF-κB/AMPK pathways [62]. More recently, limonene was found to attenuate TNF-α/IFN-γ- induced immune response in HaCaT cells by modulating MAPK, NF-κB, and JAK/STAT signaling pathways, which led to reduced expression of cytokines, including IL-6, IL-8, and IL-1β [63].

The contribution of gamma-terpinene and p-cymene cannot be ignored either. Gamma-terpinene has been shown to modulate cytokine production, such as IL-1β, IL-6, TNF-α, and IL-10 [64,65]. In parallel, para-cymene has demonstrated the ability to downregulate pro-inflammatory mediators and influence key signaling pathways, including NF-κB, ERK1/2, MAPK, and JNK pathways [66,67].

Taken together, these data suggest that AVEO’s anti-inflammatory properties are probably mediated by multiple molecular mechanisms. Its main components interact with and modulate several intracellular signaling cascades, particularly NF-κB, TLR4/MyD88, MAPK, Nrf2, NLRP3 inflammasome, and PPARα/γ. The synergy between AVEO’s components provides a convincing molecular basis for its pharmacological efficacy in inflammation-related conditions.

To gain a deeper understanding of AVEO’s action, we further investigated its anti-inflammatory properties through in vitro assays, focusing on the modulation of cyclooxygenase enzymes. Given the critical role of COX-1 and COX-2 in mediating inflammation, these enzymes were selected as primary targets to assess AVEO’s inhibitory potency and selectivity.

COX-1, a constitutively expressed enzyme, is essential for maintaining gastrointestinal integrity, while COX-2 is inducible and predominantly associated with inflammation and pain [68]. Evaluating AVEO’s selectivity and efficacy against these enzymes is crucial for elucidating its probable mechanism of action, as observed in the carrageenan-induced paw edema model.

The in vitro COX inhibition assays revealed that AVEO exhibited potent inhibitory activity against COX-2, similar to that of diclofenac, while its COX-1 inhibition was moderate compared to diclofenac and celecoxib. The selectivity index further supports this tendency, with AVEO showing an SI of 5.56, indicating a strong preference for COX-2. This compares favorably with diclofenac (SI = 3.79) but is lower than celecoxib (SI = 19.41), which is known for its COX-2 selectivity. The significant inhibition of COX-2 is particularly relevant, as this enzyme is highly expressed in inflammatory conditions. The consistency between these findings and the in vivo results suggests that its anti-inflammatory action may be, at least in parts, mediated by COX-2 inhibition. Notably, AVEO’s weaker inhibition of COX-1 may offer a therapeutic advantage, as maintaining COX-1 activity is essential for physiological functions such as gastric mucosal protection and platelet aggregation [69,70]. Thus, the preferential inhibition of COX-2 over COX-1 may reduce the risk of gastrointestinal side effects commonly associated with non-selective NSAIDs, positioning AVEO as a possibly safer alternative.

To further investigate the compounds contributing to COX-2 inhibition, we conducted in silico study to assess the binding affinities and molecular interactions of AVEO’s compounds with COX-2. Diclofenac, employed as a reference NSAID in previous experiments, was also included for comparative analysis.

The molecular docking results indicated that carvacrol and thymol exhibited a significant COX-2 inhibitory activity, with binding affinities equivalent to −7.381 and −6.939 Kcal/mol, respectively, closely matching that of diclofenac (−7.837 Kcal/mol). Both compounds demonstrated their potency by forming hydrogen bonds, hydrophobic bonds and Pi–Pi interactions with key residues at the enzyme’s active site. Specifically, they established a single H-bond with the residue Ser530 as a result of the interaction between their phenolic hydroxyl group and the Ser530 hydroxyl group. In contrast, diclofenac did not form any H-bonds. Previous studies have reported that a Ser530 mutant of COX-2 exhibited a 240-fold higher IC_50_ for diclofenac than the wild-type enzyme [71]. In another study [72], it was demonstrated that the S530A-COX-2 mutant displayed complete resistance to diclofenac inhibitory effects. Moreover, the impact of the S530A mutation on other NSAIDs, such as ibuprofen, naproxen, and mefenamic acid, was also reported, in which the mutation caused a modest 2-fold increase in the IC_50_ values for ibuprofen and naproxen, while the IC_50_ for mefenamic acid increased by 3.7-fold [72]. The differential sensitivity observed among these NSAID suggests that while Ser530 is important, the overall binding affinity is influenced and stabilized by additional interactions with other residues in the binding pocket. The residue Ser530 also represents a target for acetylation by aspirin, which inhibits the formation of prostaglandin E2 (PGE2) [73,74].

Furthermore, carvacrol and thymol established several hydrophobic bonds (Table 5). It was noted that Tyr385 is the critical catalytic residue that initiates the cyclooxygenase reaction. During activation of the enzyme, Tyr385 donates a hydrogen atom to the heme group, generating a tyrosyl radical, which then abstract the 13-pro-(S)-hydrogen atom from the arachidonic acid (AA), suggesting that a Tyr385 mutation could eliminate completely the cyclooxygenase activity [74]. Val349 and Trp387 are other examples of important residues in the COX-2 binding site, in which it has been suggested that Val349 helps to anchor the carboxyl end of the arachidonic acid while Trp387 has a role in correct positioning of the AA [74]. On the other hand, structure analysis of 5KIR shows that the COX-2 binding pocket comprises His90, Arg513, Leu352, Ala527, Ser530, Val349, Val523, Phe518, Ser353, Tyr355, and Trp387 [75]. Interestingly, the best hits interact with nearly the same residues as the native ligand, rofecoxib, and the reference drug, diclofenac, highlighting their potential for COX-2 inhibition.

Molecular dynamics simulation further validated these findings, demonstrating the stable binding behavior of carvacrol and thymol within the COX-2 active site. Both compounds maintained consistent interactions with key residues throughout the simulation, suggesting potent affinity and stability. Therefore, with prolonged simulation time, they could exhibit increased stability, which would be effective in fully understanding their binding dynamics and potential. In addition, both compounds met Lipinski’s and Veber’s rules, indicating favorable oral bioavailability. However, while these properties are promising, further studies on safety, pharmacokinetics, and efficacy remain critical for their therapeutic assessment.

The in silico results align with previous studies on COX-2 inhibition by carvacrol and thymol; [76] reported that carvacrol not only significantly inhibited COX-2 with an IC_50_ of 0.8 µM, comparable to indomethacin and NS-398 (IC_50_ = 0.7 and 0.8 µM, respectively) but also showed similar inhibition of COX-1 (IC_50_ = 0.7 µM), suggesting a non-selective inhibition mechanism. Likewise [77], found that thymol inhibited COX-2 with an IC_50_ of 1 µM. These results support AVEO’s anti-inflammatory effects, suggesting that carvacrol and thymol may be key contributors to its potent COX-2 inhibition.

Considering the close link between inflammation and pain, evaluating AVEO’s analgesic properties is important, particularly its ability to modulate nociceptive pathways. Both central and peripheral nociceptive pathways are known to be implicated in pain and inflammation. In this study, to assess AVEO’s peripheral analgesic activity, the acetic acid-induced writhing assay, a well-established model for investigating analgesic agents, was used. Intraperitoneal injection of acetic acid induces acute peritoneal inflammation characterized by tissue damage. It activates the visceral and somatic nociceptors innervating the peritoneum and promotes the local release of endogenous substances that produce nociception, including prostaglandins, prostacyclin, thromboxane, bradykinin, TNF-α, IL-1β, and IL-8 [78].

The results demonstrated that AVEO significantly and dose-dependently reduced the writhing response, suggesting a peripheral anti-nociceptive effect. Notably, at 200 mg/kg, AVEO exhibited a statistically comparable analgesic effect to that of diclofenac. This effect may be attributed to the inhibitory properties of AVEO components on arachidonic acid metabolites [79,80], particularly through its COX-2 blockade, as observed in vitro. The inhibition of this enzyme reduces the synthesis of pro-inflammatory prostaglandins, which play a crucial role in sensitizing peripheral nociceptors. By decreasing these mediators, AVEO may effectively reduce pain perception at the inflamed site, leading to diminished nociceptor activation. Moreover, the AVEO’s monoterpenoid components, such as carvacrol and thymol, are known to modulate key inflammatory mediators, including TNF-α, IL-1β, and IL-6, which are among the involved mediators in peripheral pain sensitization.

Beyond its peripheral action, AVEO also demonstrated central analgesic properties in the tail immersion test. At both doses, AVEO significantly prolonged tail withdrawal latency; specifically, at 200 mg/kg, it showed comparable efficacy to tramadol. Central analgesic agents, including opioids, tramadol, and dextropropoxyphene, typically exert their effects via opioid receptor interactions [81]. In this study, AVEO’s ability to prolong tail immersion time suggests a central pain-modulating action, likely mediated by multiple mechanisms. Among these, its anti-inflammatory activity and COX-2 inhibition may play a significant role, as COX-2 inhibition in the central nervous system reduces the synthesis of pain-stimulating prostaglandins, thereby decreasing nociceptive transmission and increasing pain tolerance in response to thermal stimuli. Moreover, AVEO may also influence the release of somatostatins, endogenous pain mediators, as well as modulate key pathways involved in spinal and supraspinal pain regulation.

Overall, these findings highlight AVEO’s dual analgesic action, targeting both peripheral and central nociceptive pathways, reinforcing its potential as a natural analgesic agent. The observed effects may be attributed to the presence of bioactive monoterpenes, whose anti-nociceptive activities are well supported by literature.

The dominant compound, carvacrol, has demonstrated significant analgesic effects across various pain models, including marked inhibition of both the neurogenic and inflammatory phases in the formalin test, as well as reductions in pain behaviors in response to thermal and chemical stimuli such as acetic acid, capsaicin, and glutamate [82,83]. Carvacrol was also shown to suppress sciatic nerve excitability and block action potential generation in dorsal root ganglion neurons by inhibiting voltage-gated sodium channels, indicating a local anesthetic-like action [84]. At the central level, it modulated glutamatergic transmission in the spinal dorsal horn by altering excitatory neurotransmitter release [85,86]. Furthermore, behavioral assays such as forced swimming and tail suspension tests have demonstrated its antidepressant-like effects, likely linked to interactions with the dopaminergic system [87]. Thymol, a structural isomer of carvacrol, also exerts local anesthetic properties indicated by its capacity to modulate neuronal excitability and inhibit pain transmission through blockade of voltage-gated sodium channels [88], as well as its capacity to suppress the excitability of rat sciatic nerves [89].

In addition, limonene has been reported to alleviate mechanical hyperalgesia in neuropathic pain models such as spared nerve injury and infraorbital nerve constriction [90,91]. These effects are associated with the suppression of pro-inflammatory signaling, notably via downregulation of TNF-α, inhibition of NF-κB and p38MAPK pathways, and interference with PKC and PKA activity [91]. Moreover, in sensory neurons, it modulated TRPA1 channels and intracellular calcium levels [92]. Additionally, reduced Fos expression in the spinal cord following limonene treatment suggests central modulation of nociceptive responses [93].

Para-cymene also demonstrated significant analgesic activity in various experimental models. It effectively reduced nociceptive responses in acetic acid-induced writhing, formalin, and hot-plate tests [94,95,96]. Its anti-nociceptive effects are mediated through both peripheral and central mechanisms, particularly involving the opioid system, as evidenced by its antagonism by opioid receptor blockers such as naloxone, naltrindole, nor-BNI, and CTOP [97,98]. Additionally, p-cymene modulated pain-related pathways by reducing spinal Fos expression while enhancing activity in the periaqueductal gray (PAG) and nucleus raphe magnus (NRM), indicating the involvement of descending pain pathways [99]. Lastly, gamma-terpinene exhibited marked analgesic properties by significantly reducing nociceptive responses in formalin, glutamate, and capsaicin-induced pain models [100]. Its mechanism of action appears to involve multiple pathways, including opioid, cholinergic, KATP, and nitric oxide systems, as indicated by the reversal of its action upon pretreatment with specific antagonists [100]. In neuropathic and cancer-induced pain models, gamma-terpinene was also found to reduce pro-inflammatory cytokines such as IL-1β and TNF-α [65,101], inhibit iNOS expression, suppress c-Fos activation in the spinal cord, and lower calcium current density [101], all of which point to its capacity in modulating both inflammatory and neurogenic components of pain.

Finally, despite the encouraging results obtained in this study, some limitations need to be acknowledged. First, AVEO’s anti-inflammatory and analgesic effects were evaluated using acute models, which, while well-established, do not fully capture the oil’s potential in chronic conditions. Future studies, including chronic models simulating chronic inflammatory conditions, or neuropathic pain, could provide a more comprehensive understanding of AVEO’s therapeutic potential. Furthermore, exploring AVEO’s effects on additional pro- and anti-inflammatory mediators, transcription factors, and oxidative stress-related targets and investigating its interaction with pain-related targets would provide insights into the pathways through which AVEO exerts its anti-inflammatory and analgesic properties. Lastly, although acute oral toxicity was conducted, it was limited to short-term observations at specific doses. Long-term toxicity studies, including sub-chronic and chronic toxicity, are required to ensure the safety of AVEO for therapeutic applications.

## 4. Materials and Methods

### 4.1. Chemicals

For the various assays conducted in this study, the following chemicals and reagents were used: DPPH (2,2-diphenyl-1-picrylhydrazyl), ABTS (2,2′-azino-bis(3-ethylbenzothiazoline-6-sulfonic acid)), potassium persulfate, potassium ferricyanide, phosphate buffer, trichloroacetic acid, deionized water, ferric chloride, sulfuric acid, sodium phosphate, ammonium molybdate, methanol, Tween-80, physiological saline, carrageenan, diclofenac sodium, ascorbic acid, DMSO (dimethyl sulfoxide), tramadol, distilled water, acetic acid, and α-tocopherol. All reagents were of analytical grade and purchased from Sigma Aldrich, LLC (Chemie, Eschenstr, 5 82024 Taufkirchen, Germany), including the standard compounds with purity percentages ranging from 98.00% to 99.90%.

### 4.2. Plant Collection and Identification

The aerial parts of *Ammoides verticillata*, consisting of leaves, flowers, and seeds, were collected from the Tlemcen region (western Algeria) at full inflorescence in May 2023. The species was identified by botanists from the Laboratory of Ecological Management of Natural Ecosystems, Tlemcen University. A voucher specimen (VP-AVS052022) was deposited in the herbarium of the Laboratory of Microbiology Applied to Agrifood, Biomedical and the Environment in the same institution. The plant material was air-dried in the dark at room temperature for 20 days.

### 4.3. Experiment Animals

Male *Wistar albino* rats, weighing 180 and 200 g, were obtained from the Pasteur Institute in Algeria and housed under standard laboratory conditions with a 12 h light/dark cycle, controlled room temperature, and free access to food and water. Prior to the commencement of any experimental procedures, a two-week acclimatization period was allowed. All methodologies were performed according to the guidelines established and approved by the local ethics committee (NCBR/0118/2023). These animal guidelines complied with the ARRIVE guidelines and those of the National Institutes of Health guide for the Care and Use of Laboratory Animals (NIH Publications No. 8023, revised 1978).

### 4.4. Extraction and Chemical Characterization of Essential Oil

The essential oil of *A. verticillata* was extracted by steam distillation of dried plant material using a Clevenger-type apparatus, following the guidelines of the European pharmacopeia [102]. The obtained EO was separated, dried over anhydrous magnesium sulfate, and stored in a dark glass at 4 °C until further analyses.

The chemical composition of *A. verticillata* EO was analyzed using gas chromatography–mass spectrometry (GC-MS). The analysis was conducted on a Perkin Elmer auto-system XL chromatograph coupled to a turbo mass spectrometer, equipped with an automatic injector, and two capillary columns with distinct polarities: a polar column (Rtx-Wax, polyethylene glycol) and a nonpolar column(Rtx-1, polydimethylsiloxane), both measuring 60 m in length, with an internal diameter of 0.22 mm and a film thickness of 0.25 μm. Helium was used as the carrier gas at a flow rate of 1 mL/min, with a column head pressure of 25 psi. The injector temperature was set to 250 °C, and 0.2 μL of EO was injected in split mode (split ratio 1:80). The oven temperature was programmed to start at 60 °C, increasing at 2 °C/min until reaching 230 °C, where it was held constant for 35 min. Detection was carried out using a quadrupole mass analyzer operating in electron impact (EI) mode at 70 eV ionization energy, with a source temperature of 150 °C. Mass spectra were recorded over a range of 35–350 Da. The identification of individual components was achieved by comparing retention indices (RIs) determined on polar and non-polar columns with those of authentic compounds and literature data [103,104,105]. Then, the compound’s identification was confirmed by comparing the mass spectra to those in our own library, which has mass spectra of real compounds and data from the literature.

### 4.5. Assessing Antioxidant Efficacy

The antioxidant properties of AVEO were evaluated using four colorimetric assays. Ascorbic acid and α-tocopherol were used as reference antioxidants for comparison. To ensure reliability, all samples were freshly prepared on the day of analysis, and the results are presented as the mean of three independent experiments.

#### 4.5.1. DPPH Radical Scavenging Assay

DPPH**^•^** radical scavenging activity was assessed following the method of [106], with minor adjustments. Different concentrations of AVEO (150 µL) were mixed with methanolic DPPH solution (60 µM, 1350 µL) and incubated in the dark for 30 min. Absorbance was measured at 517 nm against a methanol blank. The percentage inhibition of DPPH**^•^** radicals was calculated using the following formula:

I (%) = (Ac − As/Ac) × 100

where Ac represent the absorbance of the negative control and As is the absorbance of the sample.

#### 4.5.2. ABTS Radical Scavenging Assay

The ability of AVEO to neutralize the ABTS**^•+^** cation radical was evaluated following the procedure detailed by [107]. ABTS**^•+^** radicals were generated by mixing equal volumes of ABTS (7 mM) and potassium persulfate (2.45 mM), followed by incubation in the dark at 4 °C for 16 h. The resulting solution was diluted with distilled water to achieve an absorbance of 0.700 ± 0.025 at 734 nm. For the assay, AVEO (40 µL) was mixed with ABTS**^•+^** solution (160 µL) and incubated in the dark for 10 min. Absorbance was then measured at 734 nm, and the percentage inhibition was calculated as described above.

#### 4.5.3. Ferric Reducing Power Assay (FRAP)

The reducing power of AVEO was determined using the method of [108]. Briefly, AVEO (10 µL) was mixed with potassium ferricyanide (1%, 50 µL) and phosphate buffer (0.2 M, pH = 6.6, 40 µL). After incubation at 50 °C for 20 min, trichloroacetic acid (10%, 50 µL), deionized water (40 µL), and ferric chloride (FeCl_3_, 0.1%, 10 µL) were added. Absorbance was measured at 700 nm, and results were expressed as IC_50_ values, representing the concentrations required to achieve an absorbance of 0.5.

#### 4.5.4. Total Antioxidant Capacity (TAC)

The total antioxidant capacity was measured using the phosphomolybdenum method described by [109]. In brief, 100 µL of the sample solution (2 mg/mL) was mixed with 1 mL of the phosphomolybdenum reagent (0.6 M sulfuric acid, 28 mM sodium phosphate, and 4 mM ammonium molybdate) and incubated at 95 °C for 90 min. After incubation, absorbance was recorded at 695 nm. A calibration curve was prepared using ascorbic acid under the same conditions, and results were expressed as µg ascorbic acid equivalent per mg of EO (µg AAE/mg EO).

### 4.6. Acute Oral Toxicity Assessement

The acute toxicity of AVEO was evaluated in accordance with OECD 425 research guidelines [110]. Healthy male Wistar rats were randomly divided into three groups (n = 6 per group). Two doses of AVEO, 1000 mg/kg and 2000 mg/kg body weight, were selected for testing. The EO was solubilized in physiological saline containing 3% of Tween-80. Prior to dosing, animals were fasted overnight with water available ad libitum. Group 1 received 1000 mg/kg of AVEO, group 2 received 2000 mg/kg, and group 3 (control) received an equivalent volume of the vehicle (3% of Tween-80 in physiological saline), all administered orally via gavage. Post dosing, animals were observed individually for the first 30 min, then periodically over the first 24 h. Daily observations continued for 14 days to monitor any manifestations of convulsions, tremors, diarrhea, salivation, lethargy, changes in body weight, respiratory difficulties, and mortality.

At the end of the observation period, Blood samples were collected from the orbital plexus puncture using a fine heparinized capillary tube and centrifuged at 3000 rpm for 10 min at 4 °C to check the biochemical parameters, including creatinine, uric acid, total cholesterol, triglycerides, ALP, SGOT, and SGPT (serum glutamic oxyacetic transaminase and serum glutamic pyruvic transaminase), using an automatic biochemical analyzer (Hitachi 7180, Hitachi high tech corporation, Kita-ku, Tokyo, Japan); subsequently, all surviving animals were humanely euthanized.

### 4.7. Anti-Inflammatory Activity

The anti-inflammatory activity of AVEO was evaluated using the carrageenan-induced paw edema model, as described by [111]. The rats were randomly allocated into five groups (n = 5 per group). The first group served as the negative control and received normal saline (vehicle), while the second group was used as positive control and treated with sodium diclofenac (20 mg/kg) as the reference drug. The remaining three groups were administered AVEO at doses of 50, 100, and 200 mg/kg. All treatments were administered orally one hour before the subcutaneous injection of 0.1 mL of carrageenan solution (1% *w*/*v* in saline) into the plantar surface of the left hind paw. Paw edema was measured using a digital Vernier caliper before carrageenan injection (0 h) and 1, 2.5, and 5 h post-injection. The anti-inflammatory activity was quantified by determining the percentage reduction in edema using the formula:

Percentage edema = [average paw thickness (after 1, 2.5, or 5 h) − average paw thickness (0 h)]/average paw thickness (0 h).

### 4.8. Analgesic Activity

#### 4.8.1. Tail Immersion Test

This test was used to evaluate the thermal analgesic activity of AVEO according to the method of [112], with slight modifications. Thirty-five rats were randomly assigned to five groups (n = 7 per group). The control group received distilled water (10 mL/kg), the reference group (positive control) was administered tramadol (10 mg/kg), and the experimental groups were treated with AVEO at doses of 50, 100, and 200 mg/kg. Before treatment (baseline) and at intervals of 30, 60, 90, and 120 min post administration, each rat was gently restrained in a cloth, and the distal 2 cm of its tail was immersed in a thermostatically controlled warm water bath (55 ± 0.5 °C). The withdrawal latency was recorded in seconds, with a cutoff time of 15 s to prevent tissue damage. The analgesic efficacy of AVEO was evaluated by comparing tail flick latencies of treated groups with the negative control group.

#### 4.8.2. Acetic Acid-Induced Writhing Test

The writhing test was conducted to assess the anti-nociceptive potential of AVEO in chemically induced pain, following the method of [113]. Rats were randomly divided into five groups (n = 7 per group): a control group receiving normal water, a reference group treated with sodium diclofenac (20 mg/kg p.o.), and three experimental groups administered AVEO at 50 mg/kg, 100 mg/kg, and 200 mg/kg (orally). Thirty minutes after treatment, nociception was induced by an intraperitoneal injection of 1% acetic. Abdominal writhing responses was recorded for 30 min, starting 5 min post injection. The analgesic effect was expressed as the percentage inhibition of writhing compared to the control group.

### 4.9. In Vitro Cyclooxygenas-1 and Cyclooxygenase-2 Inhibition Assays

The COX-1 and COX-2 inhibition potentials of AVEO were evaluated according to the method described by [114]. Screening kit assays were performed using a fluorometric-based COX inhibitor screening kit according to the manufacturer’s instructions (Abcam, United Kingdom). The kits are provided with isoforms of COX-1 (Catalog Number: ab204698) and COX-2 (Catalog Number: ab283401). These assays utilize fluorometric detection to measure prostaglandin G2, which is produced by the cyclooxygenase enzyme through the conversion of arachidonic acid. A unique mix of arachidonic acid, NaOH, COX probe (dissolved in DMSO), and COX cofactor (dissolved in DMSO) was used for each test. The screening kit for inhibiting COX-1 features the COX-1 enzyme from sheep with the COX-1 inhibitor SC560 (dissolved in DMSO), while the kit for inhibiting COX-2 includes the COX-2 enzyme recombinant from humans and the COX-2 inhibitor celecoxib.

Different concentrations of AVEO or tested compounds (0.1–100 µg/mL) were first dissolved in DMSO and subsequently diluted with COX Assay Buffer. The inhibitor control (IC) was prepared by adding 2 µL of COX-1/COX-2 inhibitor and 8 µL of COX assay buffer to the appropriate wells. To prepare the solvent control (SC), a mixture of 2 µL of DMSO and 8 µL of COX assay buffer was combined in designated wells. The Sample (S) and enzyme control (EC) were formed by adding 10 µL of the diluted test compounds or COX assay buffer to their respective wells.

The reaction master mix was initially prepared by adding 76 µL of COX assay buffer, 1 µL of COX probe, 2 µL of diluted COX cofactor, and 1 µL of COX-1/COX-2 enzyme. After that, 80 µL of reaction mix was carefully introduced into every well, followed by adding 10 µL of a diluted solution of arachidonic acid/NaOH into each well simultaneously to start the reactions. Then, the fluorescence (*Ex*/*Em* 535/587 nm) was measured kinetically from 0 to10 min. The relative fluorescence units (ΔRFU) for each sample were determined by subtracting the RFU value at time t1 from the RFU value at time t2 (10 min). The slope for each sample was calculated by dividing ΔRFU by the time interval Δt (t1 − t2). Experiments were conducted in triplicate. The percent inhibition of COX-1/2 activity was calculated as follows:

% Relative Inhibition = (Slope of EC − Slope of S) ÷ (Slope of EC) × 100

where slope of EC is the slope of enzyme control and slope S is the slope of sample.

### 4.10. Computational Studies

#### 4.10.1. Molecular Docking

Molecular modeling studies and all preparatory steps were conducted using the Schrödinger suite (v. 11.8, Schrödinger, LLC, New York, NY, USA). The main bioactive constituents of AVEO were downloaded from the PubChem database, imported into Maestro graphical interface, and subjected to 3D protonation and energy minimization with the OPLS-2005 force field to generate stable, low-energy conformations [115]. The X-ray structure of human cyclooxygenase-2 (PDB ID: 5KIR, with a resolution of 2.70 Å) [116] was retrieved from the RCSB Protein Data Bank (RCSB PDB) and prepared using the Protein Preparation Wizard. Steps included correction of the structure, protonation at pH 7.0, and energy minimization using the OPLS-2005 force field to ensure structural stability. The COX-2 active site was defined based on the centroid of the co-crystallized inhibitor, rofecoxib, and a grid box was generated around key interacting residues [117,118].

Docking calculations were performed in extra precision (XP) mode using the Glide program (v. 11.8, Schrödinger, LLC, New York, NY, USA). Protocol validation was carried out by re-docking the native ligand into the COX-2 binding pocket [117]. The top-ranked compounds, with the highest docking scores, were selected for further analysis. Detailed post-docking visualization of protein–ligand interactions were performed using the pose viewer in Maestro.

#### 4.10.2. Prime MMGBSA Calculations

Binding free energies (ΔG_bind) of the selected compounds were estimated using the MMGBSA method, providing a quantitative measure of interaction strength and stability. Calculations were performed with the thermal_mmgbsa python script in Schrödinger software (v. 11.8, Schrödinger, LLC, New York, NY, USA), applying the OPLS-2005 force field and the VSGB solvation model [117,119].

#### 4.10.3. Drug-Likeness and ADME Prediction

Drug-likeness properties of the main compounds were evaluated using Lipinski’s and Veber’s rules via the SwissADME web server. Physicochemical properties and pharmacokinetics parameters were analyzed using the QikProp module in Maestro (v. 11.8, Schrödinger, LLC, New York, NY, USA).

#### 4.10.4. Molecular Dynamics Simulations (MD)

MD simulations were conducted to assess the stability of COX-2 complexes with the two top-ranked compounds. Simulations were carried out for 100 ns using the Desmond package in Schrödinger software (LLC, New York, NY, USA). Prior to simulation, the protein was prepared, and the hydrogen bonding network was optimized using the Protein Preparation Wizard. The complexes were then solvated in an SPC (single point charge) water box under orthorhombic periodic boundary conditions, maintaining a 10 Å distance from the box edges. The system was buffered using the System Builder tool, neutralized with NaCl (0.15 M) and minimized using the OPLS-2005 force field. During the simulation, the system’s temperature and pressure were maintained at 300 K and 1.01325 bar, respectively, using the NPT ensemble [27,120].

### 4.11. Statistical Analysis

All statistical analyses and graphical representations were performed using GraphPad Prism v8.4.0. Data are expressed as mean ± standard error of the mean (SEM) for in vivo experiments and mean ± standard deviation (SD) for in vitro assays. The results of in vitro assays were analyzed using one-way ANOVA, while in vivo data were analyzed using two-way ANOVA, followed by Tukey’s post hoc test for multiple comparisons. A *p*-value < 0.05 was considered statistically significant.

## 5. Conclusions

This study comprehensively evaluated the anti-inflammatory and analgesic potential of *Ammoides verticillata* essential oil, revealing promising pharmacological properties supported by experimental and computational evidence. GC-MS analysis identified a monoterpene-rich composition dominated by carvacrol, gamma-terpinene, para-cymene, limonene, and thymol, compounds previously reported to exert antioxidant, anti-inflammatory, and analgesic effects. AVEO exhibited good antioxidant activity, as demonstrated by its capacity to scavenge free radicals and reduce ferric ions, suggesting a role in modulating oxidative stress, a key contributor to inflammation and pain.

In terms of safety, acute oral toxicity evaluation indicated that AVEO has a low toxicity. In vivo, AVEO demonstrated significant anti-inflammatory activity, as evidenced by its dose-dependent inhibition of carrageenan-induced paw edema, suggesting efficacy in acute inflammatory conditions. In parallel, AVEO also exhibited notable analgesic effects, with a marked reduction in pain responses in both peripheral and central pain models. Mechanistically, AVEO showed significant in vitro COX-2 inhibitory activity, suggesting a capacity to suppress prostaglandin synthesis, a key pathway in inflammation and pain perception. Supporting this, molecular docking and MMGBSA calculations revealed stable interactions between AVEO’s major compounds, particularly carvacrol and thymol, and the COX-2 enzyme. Molecular dynamics simulations further validated these findings, aligning with the in vitro results. Although only COX-2 inhibition was experimentally verified, literature evidence strongly supports that AVEO’s main compounds also modulate other intracellular pathways, including NF-κB, TLR4, and PPARγ, providing additional support for the observed anti-inflammatory and analgesic effects. Altogether, these findings highlight AVEO as promising natural therapeutic candidate and warrant further investigations to assess long-term safety, optimize formulations, and confirm efficacy in chronic models.

## Figures and Tables

**Table 1:** Volatile constituents of *Ammoides verticillata* essential oil and their relative abundance.

#	Component	nRI ^a^	nRI ^b^	pRI ^c^	%
1	*alpha*-Thujene	925	922	1023	0.26
2	*alpha*–Pinene	933	931	1022	0.77
3	*beta*–Pinene	973	970	1110	0.51
4	Myrcene	982	979	1159	0.89
5	*para*-Cymene	1015	1011	1268	15.45
6	Limonene	1024	1020	1199	15.22
7	*gamma*-Terpinene	1051	1047	1243	17.59
8	Linalool	1081	1080	1544	0.22
9	(*E*)–Sabinene hydrate	1085	1083	1541	0.23
10	Camphor	1126	1123	1517	0.14
11	*Iso*-Menthone	1143	1142	1658	0.12
12	Borneol	1153	1148	1698	0.4
13	Terpinen–4–ol	1164	1161	1600	0.68
14	*alpha*–Terpineol	1179	1179	1700	0.1
15	Thymyl methyl oxide	1215	1215	1586	0.32
16	Carvacrol methyl ether	1226	1231	1603	0.24
17	Thymol	1270	1266	2189	12.49
18	Carvacrol	1283	1278	2219	32.51
Monoterpene hydrocarbons (%)					49.92
Oxygenated monoterpenes (%)					46.66
Total identified (%)					96.58

Order of elution is given on the nonpolar column (Rtx-1). (a) Retention indices for each compound calculated on the nonpolar Rtx-1 column. (b) Retention indices from the literature for the nonpolar column. (c): Retention indices for each compound calculated on the polar Rtx-wax column.

**Table 2:** In vitro evaluation of the antioxidant capacity of *Ammoides verticillata* essential oil.

	DPPH	ABTS	FRAP	TAC
	IC_50_ (μg/mL)			µg AAE/mgEO
AVEO	83.11 ± 0.34 ^bc^	3.52 ± 0.02 ^bc^	57.81 ± 1.88 ^bc^	7.689 ± 0.082
Ascorbic acid	3.45 ± 0.16 ^a^	7.43 ± 0.29 ^ac^	24.62 ± 1.20 ^ac^	-
α-Tocopherol	4.36 ± 0.68 ^a^	9.59 ± 0.08 ^ab^	37.39 ± 2.14 ^ac^	75.640 ± 0.075

The results are presented as mean SD, *n* = 3. (a) Significantly different from AVEO. (b) Significantly different from ascorbic acid. (c) Significantly different from α-tocopherol at *p* < 0.001.

**Table 3:** In vitro COX-1 and COX-2 potential of *Ammoides verticillata* essential oil compared to reference inhibitors.

Sample	IC_50_ Data (μg/mL)		
	COX-1	COX-2	SI
AVEO	8.39 ± 0.50 ^bc^	1.51 ± 0.20 ^c^	5.56
Diclofenac sodium	4.02 ± 0.60 ^a^	1.06 ± 0.38 ^c^	3.79
Celecoxib	3.30 ± 0.47 ^a^	0.17 ± 0.04 ^ab^	19.41

The results are expressed as mean ± SD. (a) Significantly different from AVEO. (b) Significantly different from diclofenac sodium. (c) Significantly different from celecoxib at *p* < 0.001.

**Table 4:** Docking scores and binding free energies of AVEO main compounds and diclofenac toward COX-2.

Compound	PubChem CID	Docking Score (Kcal/mol)	Glide Score (Kcal/mol)	MMGBSA (Kcal/mol)
Diclofenac (standard drug)	3033	−7.837	−7.837	−34.62
Carvacrol	10,364	−7.381	−7.381	−51.50
Thymol	6989	−6.939	−6.939	−42.35
*gamma*-Terpinene	7461	−6.370	−6.370	−61.64
*para*-Cymene	7463	−6.255	−6.255	−46.95
Limonene	22,311	−5.666	−5.666	−53.05

**Table 5:** Molecular interactions of AVEO main phytochemicals and diclofenac with the human COX-2 protein.

Protein-Ligand Interactions	Hydrogen Bonds (Distance)	Hydrophobic Bonds	Polar Interactions	Charged (+) Interactions	Pi-Pi Stacking
	Interacting Residues				
5 KIR-Diclofenac	-	Tyr355, Leu352, Val349, Tyr348, Phe518, Val344, Leu534, Leu531, Ala527, Val523, Met522, Phe381, Leu384, Tyr385, Trp387	His90, Ser353, Ser530	Arg120, Arg513	Tyr355
5KIR-Carvacrol	Ser530 (1.80 Å)	Leu352, Phe518, Val349, Tyr348, Ala527, Val523, Met522, Phe381, Leu384, Tyr385, Trp387	Ser353, Ser530	-	Phe518
5KIR-Thymol	Ser530 (1.93 Å)	Val349, Leu352, Phe518, Met522, Val523, Ala527, Phe381, Leu384, Tyr385, Trp387	Ser353, Ser530	-	Trp387
5KIR-*gamma*-Terpinene	-	Leu352, Phe518, Val349, Tyr348, Ala527, Val523, Met522, Phe381, Leu384, Tyr385, Trp387	Ser353, Ser530	-	-
5KIR-*para*- Cymene	-	Val349, Leu352, Phe518, Met522, Val523, Ala527, Phe381, Leu384, Tyr385, Trp387	Ser353, Ser530	-	-
5KIR-Limonene	-	Val349, Leu352, Phe518, Met522, Val523, Ala527, Phe529, Trp387, Tyr385, Leu384, Phe381	Ser353, Ser530	-	-

## Data Availability

The data related to the presented findings are available on request from the corresponding author.

## Supplementary Materials

The following supporting information can be downloaded at: https://www.mdpi.com/article/10.3390/ph18050635/s1, Figure S1: Superimposition of the docked co-crystal ligand (in green color) and the co-crystal ligand; Figure S2: 2D Diagrams of protein-ligand interaction: (A): Carvacrol-COX-2 complex; (B): Thymol-COX-2 complex; (C): Diclofenac-COX-2 complex.; Figure S3: 3D molecular interactions of the best hits and diclofenac at COX-2 active site: (A) carvacrol-COX-2 complex; (B) thymol-COX-2 complex; (C) diclofenac-COX-2 complex; Table S1: Biochemical parameters for rats treated by AVEO (acute toxicity); Table S2: Docking scores and binding energies of AVEO constituents against COX-2; Table S3: Molecular interactions of minor compounds AVEO with the COX-2 active site; Table S4: In silico prediction of drug-likenes AVEO’s main components with important physicochemical characteristics; Table S5: Predicted pharmacokinetics properties of AVEO’s main compounds.
