# Supplementary material for: Chemical Profiling and Assessment of Analgesic and Anti-Inflammatory Activity of Ammoides verticillata Essential Oil: In Vitro, In Vivo, and In Silico Studies
Source: Pharmaceuticals (Basel). 2025 Apr 27;18(5):635. doi: 10.3390/ph18050635 (PMC12114468; doi:10.3390/ph18050635)
Supplement: Supplementary file 1 [file pharmaceuticals-18-00635-s001.zip › pharmaceuticals-3572507-supplementary.pdf]

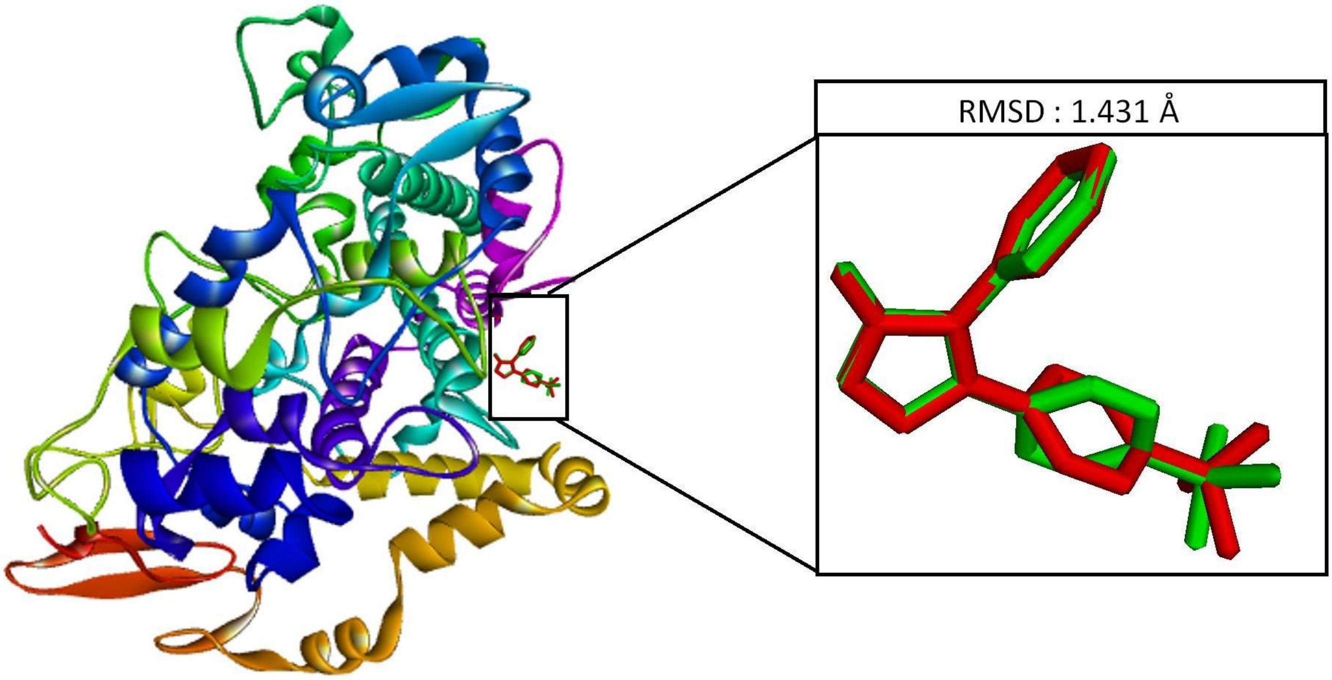

Figure S1: Superimposition of the docked co-crystal ligand (in green color) and the co-crystal ligand (in red color) with RMSD of 1.431 Å, (validation of docking study).

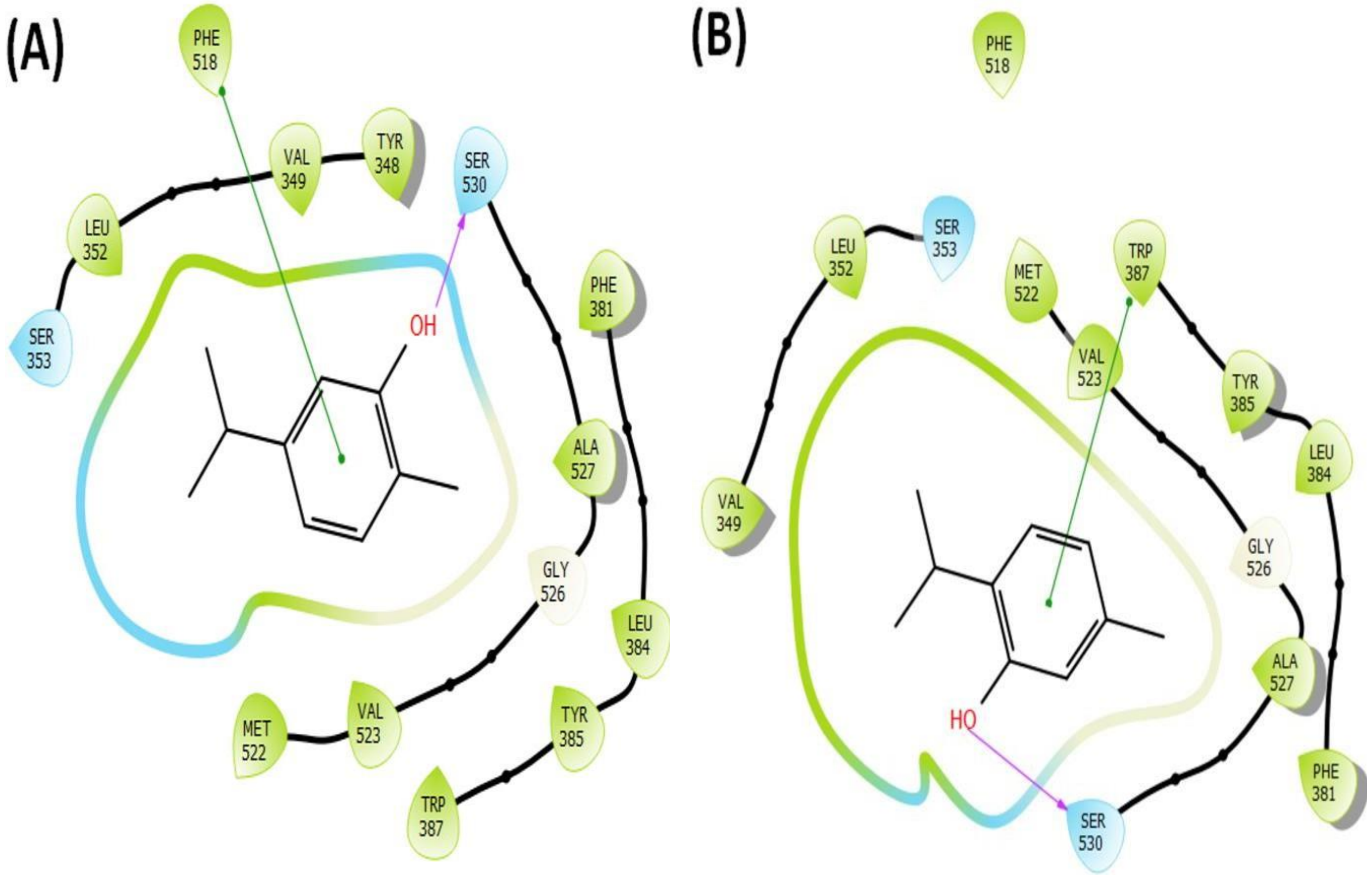

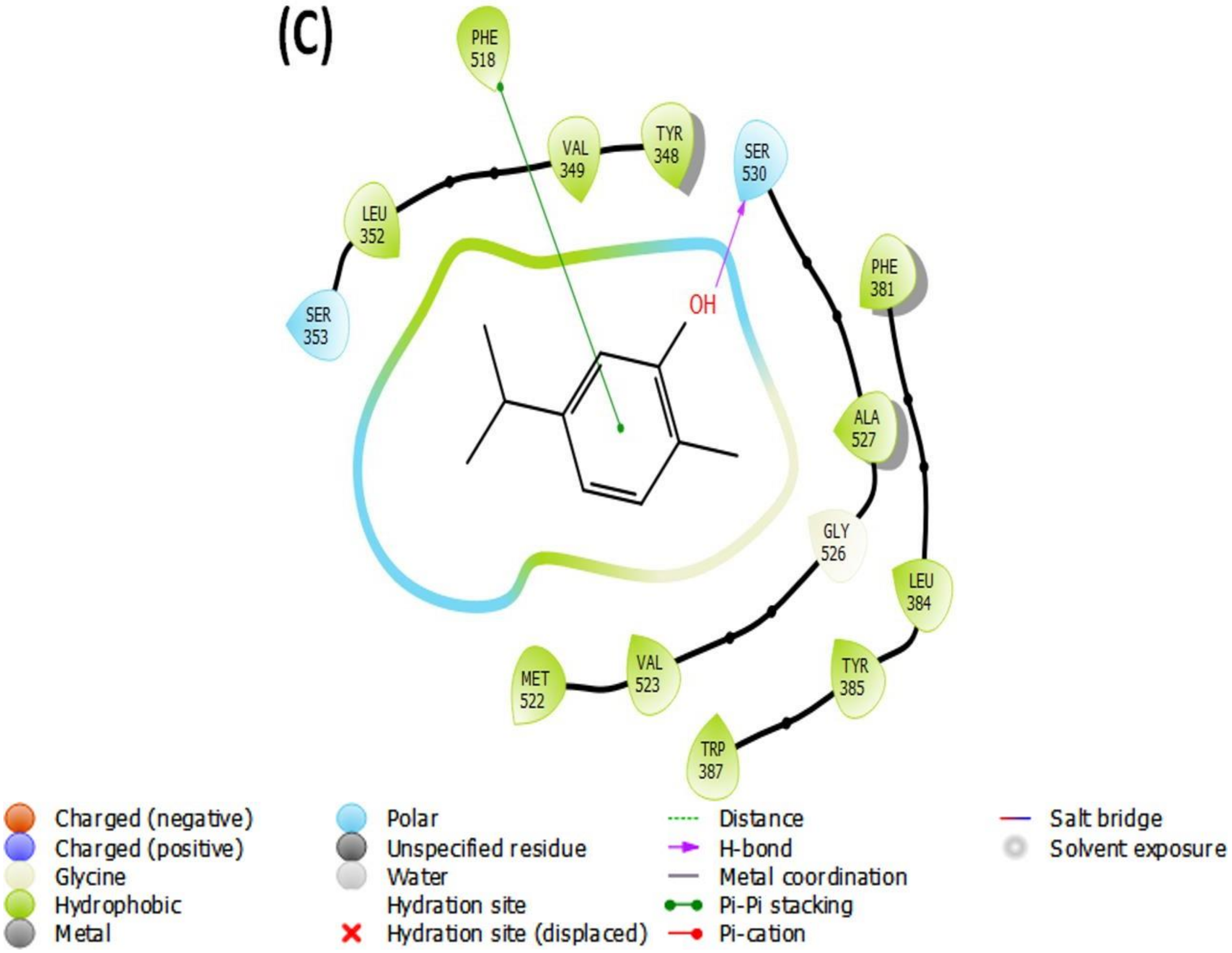

Figure S2. 2D Diagrams of protein-ligand interaction: (**A**): Carvacrol-COX-2 complex; (**B**): Thymol-COX-2 complex; (**C**): Diclofenac-COX-2 complex.

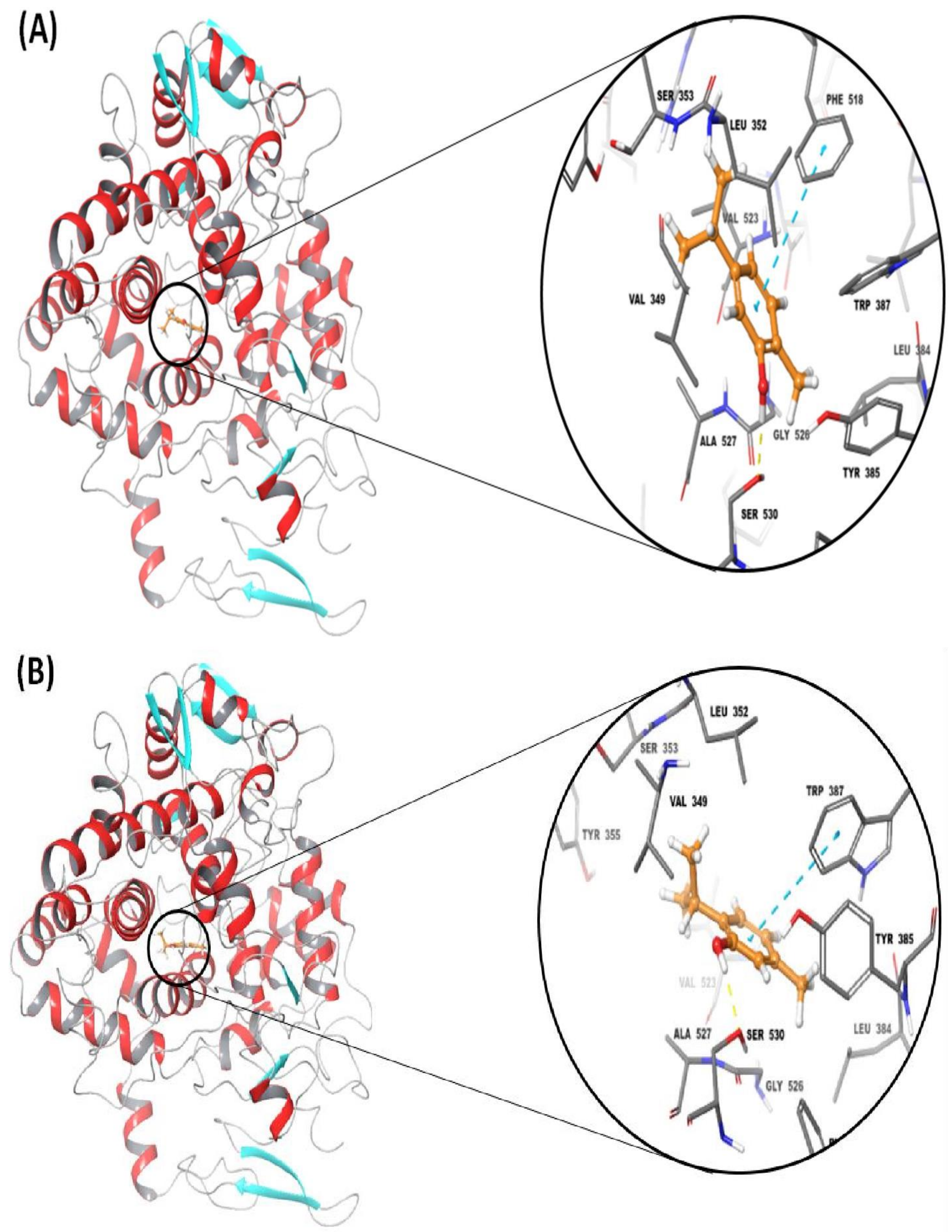

(A)
SER 353
LEU 352
PHE 518
VAL 523
VAL 349
TRP 387
LEU 384
ALA 527
GLY 526
TYR 385
SER 530
(B)
LEU 352
SER 353
VAL 349
TRP 387
TYR 355
TYR 385
VAL 523
ALA 527
SER 530
LEU 384
GLY 526

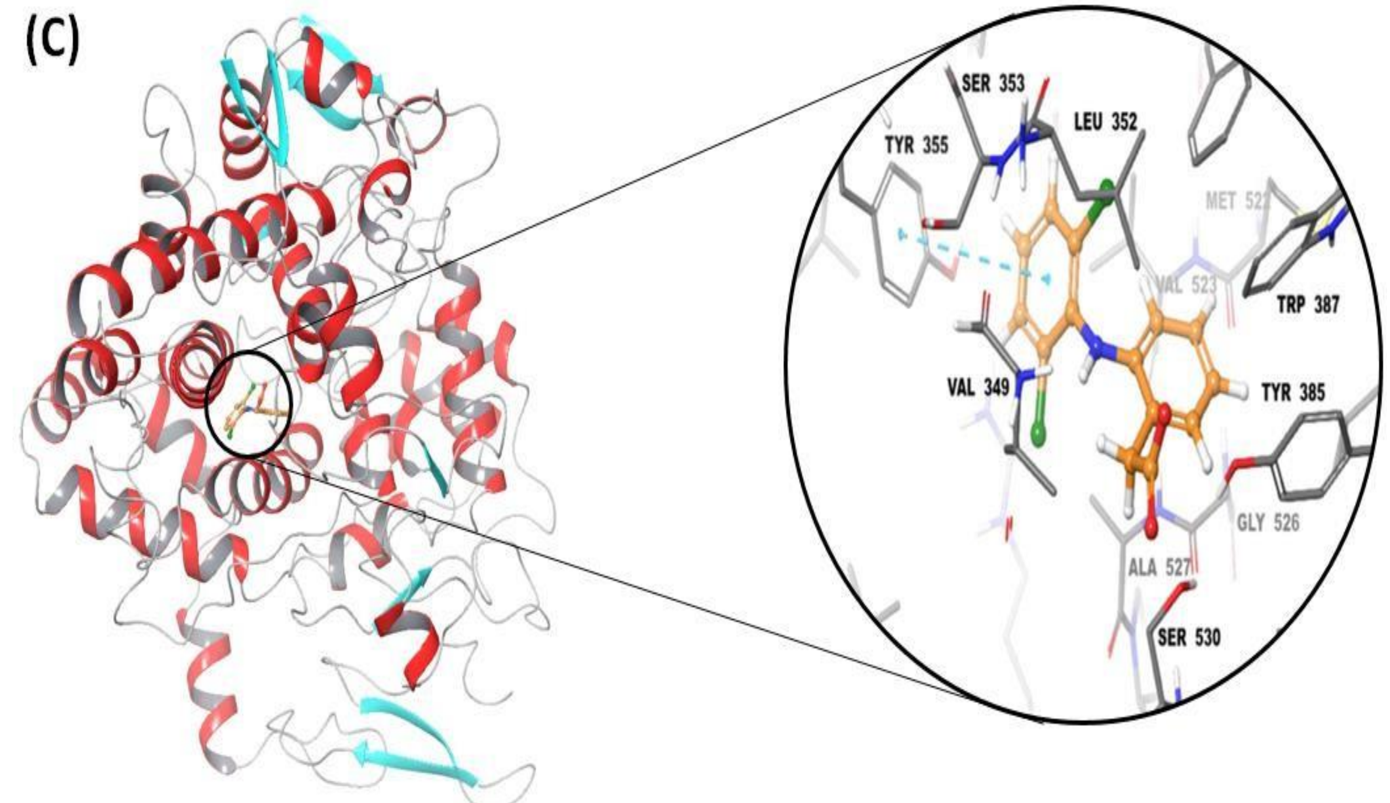

Figure S3. 3D molecular interactions of the best hits and diclofenac at COX-2 active site: (**A**) carvacrol-COX-2 complex; (**B**) thymol-COX-2 complex; (C) diclofenac-COX-2 complex.

Table S1. Biochemical parameters for rats treated by AVEO (acute toxicity).

Biochemical parameters	Group I: control	Group II: (1000 mg/kg)	Group III: (2000 mg/kg)
Triglycerides (g/l)	0.55 ± 0.06	0.48 ± 0.02	0.50 ± 0.03
Total cholesterol (g/l)	0.56 ± 0.02	0.46 ± 0.07	0.48 ± 0.02
GOT (UI/l)	51.04 ± 2.86	54.77 ± 3.48	57.17 ± 4.16
GPT (UI/l)	23.42 ± 1.26	26.83 ± 3.26	25.53 ± 1.29
ALP (UI/l)	104.90 ± 7.05	98.85 ± 3.29	112.00 ± 4.68
Uric acid (mg/l)	20.57 ± 0.93	19.38 ± 2.18	18.63 ± 1.01
Creatinine (mg/l)	4.75 ± 0.86	5.25 ± 0.25	5.73 ± 0.26

GOT (glutamic oxaloacetic transaminase/aspartate aminotransferase), GPT (glutamic pyruvic transaminase/alanine aminotransferase), ALP (alkaline phosphatase). Values are expressed as mean ± SEM.

Table S2. Docking scores and binding energies of AVEO constituents against COX-2.

Compound	PubChem CID	Docking score (Kcal/mol)	Glide score (Kcal/mol)	MMGBSA (Kcal/mol)
Diclofenac (reference inhibitor)	3033	-7.837	-7.837	-34.62
Carvacrol	10364	-7.381	-7.381	-51.50
Thymol	6989	-6.939	-6.939	-42.35
(*E*)–Sabinene hydrate	6430763	-6.640	-6.640	-56.52
Terpinen–4–ol	11230	-6.561	-6.561	-52.19
Iso-menthone	6986	-6.500	-6.500	-56.82
Carvacrol methyl ether	80790	-6.379	-6.379	-50.71
gamma–Terpinene	7461	-6.370	-6.370	-61.64
Thymyl methyl oxide	14104	-6.283	-6.283	-51.17
alpha-Thujene	17868	-6.268	-6.268	-50.08
para–Cymene	7463	-6.255	-6.255	-46.95
alpha–Terpineol	17100	-5.785	-5.785	-57.00
alpha–Pinene	6654	-5.764	-5.764	-49.93
Limonene	22311	-5.666	-5.666	-53.05
beta–Pinene	14896	-5.423	-5.423	-53.68
Camphor	2537	-5.353	-5.353	-36.47
Borneol	64685	-5.065	-5.065	-39.37
Myrcene	31253	-4.342	-4.342	-57.22
Linalool	6549	-4.239	-4.239	-52.14

Table S3. Molecular interactions of minor (remaining) compounds of AVEO with the COX-2 active site.

Protein-ligand interactions	Hydrogen bonds (distance)	Hydrophobic bonds	Polar interactions	Charged (+) interactions	Pi-Pi stacking
		**Interacting residues**			
5KIR-(E)-Sabinene hydrate	-	Tyr355, Leu352, Val349, Phe518, Met522, Val523, Ala527, Leu531, Leu359, Phe381, Leu384, Tyr385, Trp387	Ser353, Ser530	Arg120	-
5KIR-Terpinen–4–ol	Ser530 (2.50 Å)	Tyr348, Val349, Leu352, Tyr355, Leu531, Ala527, Val523, Met522, Phe518, Phe381, Leu384, Tyr385, Trp387	Ser353, Ser530	Arg120	-
5KIR-Iso-menthone	-	Tyr348, Val349, Leu352, Leu531, Ala527, Val523, Met522, Phe518, Tyr385, Trp387, Phe381	Ser353, Ser530	-	-
5KIR-Carvacrol methyl ether	-	Tyr348, Val349, Leu352, Met522, Val523, Ala527, Phe518, Phe381, Leu384, Tyr385, Trp387, Phe205	Ser353, Ser530	-	-
5KIR-Thymyl methyl oxide	-	Tyr348, Val349, Leu352, Val344, Met522, Val523, Ala527, Phe518, Phe381, Leu384, Tyr385, Trp387	Ser353, Ser530	-	Trp387
5KIR-*Alpha*-Thujene	-	Val349, Leu352, Met522, Val523, Ala527, Phe518, Phe381, Leu384, Tyr385, Trp387	Ser353, Ser530	-	-

5KIR-*alpha*–Terpineol	-	Tyr348, Val349, Leu352, Met522, Val523, Ala527, Met522, Val523, Ala527, Phe518, Phe381, Leu384, Tyr385, Trp387	Ser353, Ser530	-	-
5KIR-*alpha*–Pinene	-	Tyr348, Val349, Leu352, Met522, Val523, Ala527, Phe518, Phe381, Leu384, Tyr385, Trp387	Ser353, Ser530	-	-
5KIR-*beta*–Pinene	-	Val349, Leu352, Tyr355, Met522, Val523, Ala527, Leu531, Phe518, Tyr385, Trp387	Ser353, Ser530	Arg120	-
5KIR-Camphor	-	Tyr348, Val349, Leu352, Met522, Val523, Ala527, Leu531, Phe518, Tyr385, Trp387	Ser353, Ser530	-	-
5KIR-Borneol	-	Met522, Val523, Ala527, Leu531, Val349, Leu352, Phe518, Tyr385, Trp387	Ser353, Ser530	-	-
5KIR-Myrcene	-	Tyr348, Val349, Leu352, Met522, Val523, Ala527, Phe518, Phe381, Leu384, Tyr385, Trp387	Ser353, Ser530	-	-
5KIR-Linalool	Ser530 (2.39 Å)	Tyr348, Val349, Leu352, Tyr355, Met522, Val523, Ala527, Leu531, Phe518, Phe381, Tyr385, Trp387	Ser353, Ser530	Arg120	-

Table S4. *In silico* prediction of drug-likeness of AVEO's main components with important physicochemical characteristics.

Compound	QPlog S	QPlog $P_{o/w}$	TPSA (Å)	Lipinski rule	Veber rule	Bioavailability score
Carvacrol	-2.324	3.293	20.23	Yes	Yes	0.55
Thymol	-2.321	3.295	20.23	Yes	Yes	0.55
*gamma-*Terpinene	-4.128	4.045	0.00	Yes	Yes	0.55
*para-*Cymene	-3.689	3.648	0.00	Yes	Yes	0.55
Limonene	-4.004	3.981	0.00	Yes	Yes	0.55

QPlog S: Water solubility; QPlog $P_{o/w}$: Lipophilicity, o/w for octanol/water; TPSA: Topological polar surface area.

Table S5. Predicted pharmacokinetics properties of AVEO's main compounds.

Compound	% HO absorption (<25% is poor) (>80% is high)	Caco-2 permeability (nm/sec)	QPlogIC$_{50}$hERG (Concentration below -5)	QPlogKp (cm/h)	QPlog BB (-3 to 1.2)	CNS activity (-2 to +2)	QPlogKhasa (-1.5 to 1.5)
Carvacrol	100	3709	-3.578	-1.813	0.075	+1	0.046
Thymol	100	3835	-3.576	-1.779	0.088	+1	0.043
gamma-Terpinene	100	9906	-3.333	-1.229	0.863	+2	0.388
para-Cymene	100	9906	-3.695	-0.969	0.700	+2	0.336
Limonene	100	9906	-3.262	-1.203	0.840	+2	0.380

HO absorption: for human oral absorption; Caco-2 permeability: for Caco-2 cells permeability, a model for human intestinal absorption; QPloghERG: Predicted IC$_{50}$ value for the human-ether-a-go-go related gene (hERG) potassium channels blockage; QPlogKp: Predicted skin permeability; QPlog BB: Blood-brain coefficient; CNS activity: For central nervous system activity; QPlogKhasa: Predicted human serum albumin binding.
